# Supplementary material for: Nonlinear variations and drivers of vegetation NPP on the Tibetan Plateau: Interaction of natural and human factors
Source: PLoS One. 2025 Jul 1;20(7):e0320370. doi: 10.1371/journal.pone.0320370 (PMC12212555; doi:10.1371/journal.pone.0320370)
Supplement: S1 Appendix — (DOCX) [file pone.0320370.s001.docx]

**GEE code**

**The GEE code used for this analysis is available at the following link:**

<https://code.earthengine.google.com/919eb79ace5bfc2afb2d7fa779cc51d2>

<https://code.earthengine.google.com/f4eb6dfeebe32dd29a5e3fce0fb08bf6>

<https://code.earthengine.google.com/8647e3af3508ca5ca5deaa2c3ec6c134>
