# Supplementary material for: Nonlinear variations and drivers of vegetation NPP on the Tibetan Plateau: Interaction of natural and human factors
Source: PLoS One. 2025 Jul 1;20(7):e0320370. doi: 10.1371/journal.pone.0320370 (PMC12212555; doi:10.1371/journal.pone.0320370)
Supplement: S2 Appendix — (DOCX) [file pone.0320370.s002.docx]

**MATLAB code**

MATLAB code that calculates the coefficient of variation (CV), Hurst exponent, Sen's slope, and performs the Mann-Kendall (M-K) trend test for vegetation NPP from 2001 to 2021:

% Load NPP data (assuming nppData is a matrix with NPP values for each year)

% nppData should be a matrix where rows represent different locations and columns represent the years 2001-2021.

% Example: Load nppData (substitute with actual data loading code)

nppData = rand(100, 21); % Random example data (100 locations, 21 years)

% Year range

years = 2001:2021;

numYears = length(years);

**%% Coefficient of Variation (CV)**

meanNPP = mean(nppData, 2); % Mean NPP for each location

stdNPP = std(nppData, 0, 2); % Standard deviation for each location

CV = stdNPP ./ meanNPP; % Coefficient of Variation (CV)

disp('Coefficient of Variation (CV) for each location:');

disp(CV);

**%% Hurst Exponent Calculation**

% Using the 'hurst' function from MATLAB's Econometrics Toolbox (replace with custom code if needed)

hurstExponent = zeros(size(nppData, 1), 1);

for i = 1:size(nppData, 1)

hurstExponent(i) = hurst(nppData(i, :)');

end

disp('Hurst Exponent for each location:');

disp(hurstExponent);

**%% Sen's Slope Estimator**

% Function to calculate Sen's slope for each location

senSlope = zeros(size(nppData, 1), 1);

for i = 1:size(nppData, 1)

senSlope(i) = sen_slope(nppData(i, :), years);

end

disp('Sen''s Slope for each location:');

disp(senSlope);

**%% Mann-Kendall Trend Test**

% Use the 'mann_kendall' function to perform the M-K test

pValues = zeros(size(nppData, 1), 1);

for i = 1:size(nppData, 1)

[tau, pValue] = mann_kendall(nppData(i, :), years);

pValues(i) = pValue;

end

disp('Mann-Kendall p-values for each location:');

disp(pValues);

%% Supporting Functions

% Sen's Slope Estimator Function

function slope = sen_slope(data, years)

numYears = length(years);

slopes = [];

for i = 1:numYears-1

for j = i+1:numYears

slopes(end+1) = (data(j) - data(i)) / (years(j) - years(i)); %#ok<AGROW>

end

end

slope = median(slopes);

end

% Mann-Kendall Trend Test Function

function [tau, pValue] = mann_kendall(data, years)

% Mann-Kendall Trend Test

numYears = length(years);

s = 0;

varS = 0;

for i = 1:numYears-1

for j = i+1:numYears

s = s + sign(data(j) - data(i));

end

end

% Calculate the variance of S

varS = numYears * (numYears-1) * (2*numYears+5) / 18;

% Calculate the Z statistic

if s > 0

z = (s - 1) / sqrt(varS);

elseif s < 0

z = (s + 1) / sqrt(varS);

else

z = 0;

end

% Calculate p-value

pValue = 2 * (1 - normcdf(abs(z), 0, 1)); % Two-tailed test

tau = s / (numYears * (numYears-1) / 2); % Kendall's Tau coefficient

end
