# Supplementary material for: Nonlinear variations and drivers of vegetation NPP on the Tibetan Plateau: Interaction of natural and human factors
Source: PLoS One. 2025 Jul 1;20(7):e0320370. doi: 10.1371/journal.pone.0320370 (PMC12212555; doi:10.1371/journal.pone.0320370)
Supplement: Attachment — Submitted filename: Response to Reviewers.docx [file pone.0320370.s004.docx]

**Response to Reviewers**

**Thanks reviewers and editor for your comments, which will help improve the quality of paper.**

**ACADEMIC EDITOR:**

**I fully agree with both reviewers' comments. This manuscript is poorly organized and a large revision is needed.**

**Revised:**Thanks ACADEMIC EDITOR for your comments. We have revised this part and the revised parts has been incorporated into the paper and marked in red.

**Journal Requirements:When submitting your revision, we need you to address these additional requirements.**

**Revised:**Thanks ACADEMIC EDITOR for your comments. We have made revisions in accordance with the additional requirements.

**Reviewer #1: The research content of the article is relatively complete and has certain value for understanding the factors influencing NPP variation in the Tibetan Plateau, but there are still some shortcomings.**

**1.The article contains numerous writing errors. I advise the author to check and correct carefully. For example, an extra ":" appears in both Equations (7) and (8), and Equation (3) is incomplete. Additionally, the text references "As illustrated in Fig. 4," yet the subsequent description corresponds to the content of Fig. 3.**

**Revised:** Thanks reviewers and editor for your comments. We have revised this part and the revised parts has been incorporated into the paper and marked in red.

I advise the author to check and correct carefully. For example, an extra ":" appears in both Equations (7) and (8), and Equation (3) is incomplete. Additionally, the text references "

**Revised:** The formula for calculating Sen's trend estimation is as follows:

$\beta=median\frac{NPP_{j}-NPP_{i}}{j-i}$, 2000 i ≤ j ≤ 2020	(7)

The calculation formula for the Mann-Kendall trend test is as follows:

$S=\sum_{j=1}^{n-1} \sum_{i=j+1}^{n} \mathrm{sgn}\text{(}NPP_{j}-NPP_{i}\text{)}$ (8)

The principles underlying the calculation of the Hurst index are as follows:

(1) Given Time Series

Let the time series be {NPP(t)}, where t = 1, 2, …, n.

(2)Compute the mean.

The mean of the time series is calculated as:

$\overline{NPP_{\text{(}T\text{)}}}=\frac{1}{T}\sum_{t=1}^{T} NPP_{\text{(}T\text{)}}$ *T*= 1，2，…，*n*	(2)

(3) Construct the cumulative deviation Series

The cumulative deviation series *X*_(_*_x,T_*_)_ represents the cumulative deviation of the series from its mean:

$X_{\text{(}t,T\text{)}}=\sum_{t=1}^{T} \text{(}\mathrm{NPP}_{\text{(}t\text{)}}-\overline{NPP_{(T)}}\text{)}$ ≤ *t* ≤ *T*	(3)

(4) Calculate the range series:

The range *R*_(_*_T_*_)_ is the difference between the maximum and minimum of the cumulative deviation series:

$R_{\text{(}T\text{)}}=\max\text{ X}_{\text{(}t\text{,}T\text{)}}-\min X_{\text{(}t,T\text{)}}$ *T*= 1，2，…，*n*	(4)

(5) Calculate the standard deviation series:

The standard deviation *S*_(_*_T_*_)_ of the original time series is computed as:

$S_{\text{(}T\text{)}}=\left[\frac{1}{T}\sum_{t=1}^{T} \text{(}\mathrm{NPP}_{\text{(}t\text{)}}-NPP_{\text{(}T\text{)}}2\text{)}\right]^{\frac{1}{2}}$ *T*= 1，2，…，*n*	(5)

(6) Calculate the Hurst index:

The Hurst index *H* is estimated using the following relationship:

$H=\frac{\log\text{(}R/S\text{)}_{n}-a}{\log\text{(}n\text{)}}$ (6)

**As illustrated in Fig. 4," yet the subsequent description corresponds to the content of Fig. 3.**

**Revised:**

As illustrated in Fig. 3, the trends over time are as follows: In 2001, the highest recorded NPP was 1905 gC m^-2^.a^-1^, predominantly distributed in the southeastern portion of the plateau, reflecting robust vegetation growth during that period (Fig. 3a).

**2. The number of decimal places is inconsistent throughout the article, such as in Table 2.**

**Revised:** Thanks reviewers and editor for your comments. We have revised this part and the revised parts has been incorporated into the paper and marked in red.

**Tab.2 Statistics of climate zones and vegetation NPP on the Qinghai-Tibet Plateau from 2001 to 2021 (Unit: gC m^-2^.a^-1^)**

| **Secondary zoning code** | **Name** | **Mean** | **STD** |
| --- | --- | --- | --- |
| ⅡC5 | Yining Region | 94.85 | 38.50 |
| ⅢD1 | Southern Xinjiang Region | 109.14 | 9.61 |
| HD2 | Northern Tibet Region | 50.10 | 30.28 |
| HC3 | Southern Tibet Region | 117.34 | 106.05 |
| HC2 | Central Tibet Region | 86.69 | 51.33 |
| ⅢB3 | Weihe Region | 552.99 | 129.06 |
| ⅣA2 | Qinba Region | 641.13 | 186.27 |
| VA3 | Sichuan Region | 517.28 | 227.78 |
| HB2 | Chamdo Region | 225.03 | 93.68 |
| HA1 | Bomi-Western Sichuan Region | 342.50 | 173.76 |
| HV1 | Dawang-Zayu Region | 551.48 | 327.60 |
| HC1 | Qilian-Qinghai Lake Region | 211.03 | 120.60 |
| HB1 | Southern Qinghai Region | 132.69 | 69.29 |
| HD1 | Qaidam Basin Region | 132.96 | 62.18 |
| ⅡC2 | Central Mongolia Region | 361.98 | 124.20 |
| VA5 | Northern Yunnan Region | 784.65 | 260.93 |

**3. Fig.3 lacks annotations, which affects its readability. Please add what panels (a), (b), (c) and (d) represent, respectively.**

**Revised:** Thanks reviewers and editor for your comments. We have revised this part and the revised parts has been incorporated into the paper.

Fig.3 Vegetation NPP spatial pattern changes in the Qinghai-Tibet Plateau from 2001 to 2021

**4. The article inconsistently references the model used. Section 3.2.5 describes it as the "OPQD" model, while elsewhere it is referred to as "OPGD." Additionally, the full name is presented as the "Optimal Parameters-based Geographical Detector (OPGD) model," but the keywords and abstract list it as the " the optimized Geographical Detector (OPGD)." Please ensure consistent terminology throughout the article.**

**Revised:** Thanks reviewers and editor for your comments. We have revised this part and the revised parts has been incorporated into the paper and marked in red. The optimized Geographical Detector (OPGD) has been correctly revised based on the writing errors in Section 3.2.5, where "OPQD" was corrected to "OPGD." Similarly, the writing errors involving "OPQD" in the references have also been amended accordingly.

2.3.5 **OPGD**

(1) Parameter Optimization

The OPGD

(2) Spatial Differentiation Analysis

The OPGD

(3) Multifactor interaction analysis

The OPGD

Guo, X., Liu, Y., Zhang, Y. 2020. Analysis of vegetation NPP spatial heterogeneity in the Tibetan Plateau using OPGD model. Remote Sensing, 12(5), 743. https://doi.org/10.3390/rs12050743

Song, X., Zhang, Y., Wang, G. (2020). A new approach to spatial heterogeneity analysis: OPGD model application. Environmental Monitoring and Assessment, 192(4), 245. <https://doi.org/10.1007/s10661-020-8140-4>

Wang G, Li Y, Zhang, H. 2016. Exploring the interactions between environmental variables and vegetation: Insights from OPGD. Global Ecology and Conservation, 7, pp.64–75.

**5.Many conclusions in the article appear casual and lack supporting data or references. For instance: “These fluctuations may be closely related to interannual climate variations, changes in precipitation and temperature, as well as the impacts of human activities such as grazing and land use,” “This enhancement is likely due to favorable climatic shifts, such as increased precipitation and warmer temperatures, fostering an expansion of vegetated areas (Fig. 3b),” and “indicating that areas with higher vegetation productivity are more influenced by environmental factors. This may be related to terrain, precipitation, and human activities.” Please provide data or references to support these statements.**

**Revised:** Thanks reviewers and editor for your comments. We have provided references to support these statements, the revised parts has been incorporated into the paper and marked in red.

“These fluctuations may be closely related to interannual climate variations, changes in precipitation and temperature, as well as the impacts of human activities such as grazing and land use [15]”

15.Zhang G, Yao T, Chen F. 2020. Response of alpine vegetation to climatic changes on the Tibetan Plateau: A synthesis of remote sensing and modeling analysis. Science of the Total Environment, 703, 134979, pp. 1–15.

DOI: 10.1016/j.scitotenv.2019.134979.

This enhancement is likely due to favorable climatic shifts, such as increased precipitation and warmer temperatures, fostering an expansion of vegetated areas [60,20].

20.Zhang Y, Wang J, Li X. 2014. Spatial distribution and temporal trends of net primary productivity in the Tibetan Plateau. Remote Sensing of Environment, 154, pp. 126–138.

60.Wu C, Chen J, Shen, M, Tang, Y. 2020. Assessing vegetation dynamics and their responses to climate change on the Tibetan Plateau: A focus on net primary productivity. Science of the Total Environment, 717, 137231.

https://doi.org/10.1016/j.scitotenv.2020.137231

, indicating that areas with higher vegetation productivity are more influenced by environmental factors. This may be related to terrain, precipitation, and human activities[8,54] (Table 3, Fig 4).

8.Xu X, Zhang X, Li R. 2022. Climate variability and its impact on vegetation dynamics across the Tibetan Plateau over the past two decades. Environmental Research Letters, 17(4), 045001. DOI: 10.1088/1748-9326/ac5f30.

54.Liu J, Ji Y, Zhou G, Zhou L, Lyu X, Zhou Meng, Z. 2022. Temporal and spatial variations of net primary productivity (NPP) and its climate driving effect in the Qinghai-Tibet Plateau, China from 2000 to 2020. Chinese Journal of Applied Ecology, 33(6), pp.1533–1538.

**6. Section 4.3, titled "Vegetation NPP variation in different climate zones," discusses differences in NPP across different climate zones. However, the text describes this as "the relationship between mean and variance reveals the impact of climate and topography on vegetation productivity." It is unclear how the impact of topography on NPP is reflected in this section.**

**Revised:** Thanks reviewers and editor for your comments. We have provided references to support these statements, revised this part and the revised parts has been incorporated into the paper and marked in red.

and the relationship between mean and variance reveals the impact of climate and topography on vegetation productivity[61]. The impact of topographic complexity on vegetation productivity in different climatic zones. Plateau topography plays a significant role in vegetation productivity by influencing climate, hydrological processes, and local environments [62]. Under varying topographical conditions, differences in factors such as climate change and water supply lead to spatial disparities and variability in NPP [63]. The interaction between topography and climate manifests differently across regions, and understanding this mechanism is crucial for comprehending the spatiotemporal changes in vegetation productivity [61].

61.Liu X, Zhang F, Chen M. 2023b. Topographic control of vegetation productivity across climate zones in the Tibetan Plateau: Implications for ecosystem services under climate change. Environmental Research Letters, 18(5), 054019. https://doi.org/10.1088/1748-9326/abf300

62.Liu X, Zhang Y, Wang J, Li Q. 2023a. The impact of topographic complexity on vegetation productivity in different climatic zones. Ecological Processes, 12(3), pp.345–358.

63.Chen L, Wang X, Li, S. 2022. Effects of topography and climate on vegetation growth and productivity in the Tibetan Plateau: A spatiotemporal analysis. Global Change Biology, 28(1),pp.184–196. https://doi.org/10.1111/gcb.15930

**A more detailed explanation:** a more detailed explanation of how topography influences NPP is as follows, but due to space constraints, it has not been incorporated into the main text.

Topography influences local climatic conditions (such as temperature and precipitation), leading to climatic variations across different elevation zones, which in turn have varying impacts on vegetation productivity. For instance, alpine regions experience limited vegetation growth due to high altitudes, cold climates, and scarce precipitation, whereas lower mountainous areas benefit from warmer and more humid conditions, resulting in higher vegetation productivity (Zhang et al., 2019). Studies have shown that topographic complexity (e.g., mountains, valleys) creates localised climatic variations within different climatic zones, which further influence the spatial variability of NPP. For example, valleys facilitate the accumulation of moisture, promoting vegetation growth, while alpine areas experience suppressed vegetation productivity due to reduced precipitation and lower temperatures, causing greater fluctuations in NPP in certain regions (Li et al., 2020).

Moreover, topography affects water movement and retention, thereby determining regional water availability. The slope and aspect of mountainous areas influence precipitation accumulation and runoff, subsequently affecting soil moisture conditions, which are a critical factor for vegetation growth. Thus, variations in topography directly determine the spatial distribution of vegetation productivity (Chen et al., 2018). In regions with low NPP, such as northern Tibet and southern Xinjiang, the average NPP is lower and exhibits minimal variation, primarily due to the constraints of cold climates. Additionally, the high-altitude topographic factors further limit vegetation growth. Meanwhile, as these regions experience relatively minor climatic changes, the variability in NPP remains low, and vegetation productivity is relatively stable, displaying limited spatial fluctuation (Wang et al., 2021).

Chen, H., Zhang, W., & Peng, C. (2018). Effects of terrain on soil moisture and vegetation growth: A regional analysis. Catena, 162, 223-234. DOI: 10.1016/j.catena.2017.10.006.

Li, Y., Chen, X., & Ma, J. (2020). Influence of topographic variation on NPP spatial heterogeneity under changing climate conditions. Environmental Research Letters, 15(4), 045006. DOI: 10.1088/1748-9326/ab7d9f.

Wang, F., Gao, Y., & Xu, Y. (2021). Spatiotemporal variability of NPP in relation to topography and climate on the Tibetan Plateau. Science of the Total Environment, 753, 142206. DOI: 10.1016/j.scitotenv.2020.142206.

Zhang, L., Wang, Y., & Luo, Z. (2019). Topographic effects on vegetation productivity in mountainous regions: A case study in the Tibetan Plateau. Journal of Mountain Science, 16(5), 982-995. DOI: 10.1007/s11629-019-5427-8.

**7. The discussion section is too much and should be made further concise. In addition, some text in the results section, such as analysis of specific results and their potential causes, would be more appropriately placed in the discussion section.**

**Revised:** Thanks reviewers and editor for your comments. We have revised discussion section and the revised parts has been incorporated into the paper and marked in red.

**4.2 Natural Factors**

**4.2.1 Elevation**

Vegetation NPP varies with elevation, reflecting the ecological differences across the Tibetan Plateau. This study, based on 50 m elevation intervals, used GEE to illustrate NPP changes with altitude (Fig 9). As shown in Fig 9, NPP negatively correlates with elevation; as altitude increases, NPP decreases. This is linked to environmental factors like temperature, oxygen levels, and moisture availability [2].

At 500 m, the mean NPP is 145.017 gC m².a^-1^, the highest in the study area (Fig 9). As elevation increases, NPP drops significantly to around 83.041 gC m².a^-1^ at 2000 m, indicating a decline in productivity. Between 1500 and 3000 m, the decline is gradual (Fig 8). Above 3000 m, the decrease accelerates, particularly above 4000 m, where NPP sharply declines with every 500 m increase, falling from 30.169 gC m².a^-1^ to about 0.34 gC m².a^-1^, indicating near-zero productivity (Fig 9). At elevations near 7000 m, NPP is almost zero, showing the extreme limitations of high-altitude environments on vegetation growth [69,70].

Therefore, lower-altitude areas benefit from more favorable climatic and soil conditions, supporting vegetation growth, while higher altitudes pose increasing challenges to vegetation productivity.

2.Piao S, Fang J, Zhou L. 2003. Changes in vegetation activity in the Tibetan Plateau: Implications for ecosystem services. Global Change Biology, 9(9), pp.1253–1263.

69.Xu, J., Melick, D. 2007. The impact of climate change on vegetation dynamics in the Tibetan Plateau. Journal of Arid Environments, 68(1), pp.1–17.

70.Ni J, Zhang X, Scurlock J. M. 2013. Modeling net primary productivity of terrestrial ecosystems in China with a process-based dynamic vegetation model. Ecological Modelling, 169(1), pp.395–408.

**4.2.2 Total solar radiation**

The Tibetan Plateau, sensitive to global climate change, is strongly influenced by total solar radiation, which drives photosynthesis and vegetation NPP[71]. Due to its high altitude, low temperatures, and unique monsoon climate, the impact of solar radiation on NPP shows distinct patterns [72,73]. The partial correlation coefficient between NPP and solar radiation ranges from -0.89 to 0.85, indicating significant spatial variation with both positive and negative correlations (Fig 10a).

As shown in Fig 10a, 34.16% of the area shows a positive correlation (partial correlation coefficient > 0). Among these, 7.90% have coefficients between 0.3 and 0.6, 0.36% exceed 0.6, and 25.89% are between 0 and 0.3. Solar radiation notably enhances NPP, particularly in regions with strong radiation and favorable temperature and moisture, like southwestern Qinghai, northern Sichuan, and eastern Tibet [72,73,74,70].

In contrast, 65.84% of the area shows a negative correlation (partial correlation coefficient < 0), where increased solar radiation suppresses NPP. Areas with coefficients less than -0.6, between -0.6 and -0.3, and between -0.3 and 0 represent 2.45%, 24.09%, and 39.30%, respectively. This negative correlation is likely due to high temperatures that reduce photosynthetic efficiency and increase water evaporation, hindering plant growth. These areas are mostly found in northeastern, eastern, and southwestern Qinghai, and parts of Tibet.

71.Zhao W, Zhang Y, Wang H, Yu G. 2010. The impact of climate and land cover changes on net primary productivity in the Tibetan Plateau. Journal of Geophysical Research: Atmospheres, 115(D8), D08107. https://doi.org/10.1029/2009JD012600

72.Piao S, Wang X, Ciais P, Zhu B, Wang T, Liu J. 2011. Changes in satellite-derived vegetation growth trend in temperate and boreal Eurasia from 1982 to 2006. Global Change Biology, 17(10), pp.3228–3242.

73.Zhou L, Sun Y, Wang H. 2016. Relationship between solar radiation and vegetation net primary productivity on the Tibetan Plateau. Journal of Arid Environments, 135,pp. 1–12.

74.Wang G, Zhou H, Wang Y. 2011. Spatial variation in the relationship between solar radiation and vegetation net primary productivity in the Qinghai-Tibet Plateau. Ecological Indicators, 11(4), pp.961–969.

**4.2.3 Annual average temperature**

Partial correlation analysis shows that the correlation between vegetation NPP and annual average temperature on the Tibetan Plateau ranges from -0.80 to 0.89, with both positive and negative correlations (Fig 10b). This aligns with studies highlighting temperature's importance for plant growth but showing significant regional variation [75,76], contrasting with conclusions suggesting a general positive correlation without considering spatial heterogeneity [77].

Positive correlation: As shown in Fig 10b, 31.84% of the area shows a positive correlation (partial correlation coefficient > 0), with 30.02% having coefficients between 0.3 and 0.6 and 1.82% greater than 0.6. Temperature significantly promotes NPP in areas mainly in northeastern Qinghai, Tibet, and Sichuan. However, 52.54% of the area has a weaker effect (0 to 0.3), indicating temperature is not the primary driver of NPP variation in these regions, which are mainly in southern Qinghai, northwestern Sichuan, and Tibet.

Negative correlation: The negative correlation areas (Fig 10b) suggest that rising temperatures suppress NPP. Regions with coefficients below -0.5 account for 0.11%, and between -0.5 and -0.2, 2.98%, indicating high temperatures inhibit growth due to moisture loss. These areas are mainly in southwestern Tibet and northwestern Sichuan. Additionally, 12.53% of the area has a minor negative effect (between -0.2 and 0), suggesting other factors, such as precipitation or solar radiation, may play a larger role in NPP variation [10,78].

10.Piao S, Wang X, Ciais P. 2013. Impacts of climate change on vegetation dynamics in the Tibetan Plateau. Global Change Biology, 19(1), pp. 230–242.

75.Piao S, Wang X, Ciais P. 2006. The influence of carbon dioxide concentration on the productivity of the Tibetan Plateau. Nature, 442(7102), pp.1023–1025.

76.Wang G., Li X., Wang Y. 2014. The relationship between temperature and vegetation NPP in the Tibetan Plateau. Climate Dynamics, 42(1–2), pp.173–182.

77.Liu Z., Zhao X., Zhang L., Gao B. 2022a. The synergistic impacts of human activities and natural factors on NPP: A meta-analysis. Environmental Research Letters, 17(7), 074012. https://doi.org/10.1088/1748-9326/ac7bd9

78.Zhao M, Running S. W. 2005. Drought effects on vegetation growth in China. Remote Sensing of Environment, 94(3), pp.331–340.

**4.2.4 Accumulated temperature above 0°C**

The partial correlation coefficient between vegetation NPP and accumulated temperature above 0°C on the Tibetan Plateau ranges from -0.96 to 0.74, showing significant regional variation (Fig 10c).

Positive correlation: Positive correlation areas account for 15.22% of the total area. Of these, regions with coefficients between 0.2 and 0.5, and 0.5 to 0.74, make up 4.23% and 0.16%, respectively. These areas, mainly in the eastern and southeastern Tibetan Plateau and around the Sichuan Basin, show that increased accumulated temperature boosts plant photosynthesis, enhancing NPP under warmer conditions [11,12,79].

Negative correlation: Negative correlation areas cover 84.78% of the total area. Specifically, areas with coefficients below -0.7, between -0.7 and -0.4, and between -0.4 and 0 make up 6.91%, 27.21%, and 50.66%, respectively. In these regions, higher accumulated temperature increases evapotranspiration, reducing plant growth, particularly in the highlands of the Tibetan Plateau[7,80]. While moderate accumulated temperature promotes NPP, excessively high temperatures or water shortages hinder growth [81].

7.Zhou H, Zhao X, Zhang W. 2020. The impact of climate change on net primary productivity in the Tibetan Plateau. Journal of Arid Land, 12(4), pp. 470–482.

11.Sun J, Qin X. 2020. Precipitation dominates the interannual variability of vegetation dynamics in the Tibetan Plateau. Agricultural and Forest Meteorology, 291, 108091, pp. 1–9. DOI: 10.1016/j.agrformet.2020.108091.

12.Chen C, Zhang L, Wu Z. 2021. Effects of accumulated temperature on vegetation NPP in the Tibetan Plateau. Journal of Environmental Management, 282, 111947. DOI: <https://doi.org/10.1016/j.jenvman.2020.111947.>

79.Wang Z, He H, Li S. 2019. Spatial-temporal patterns of vegetation productivity in relation to climate variability in the Tibetan Plateau. Journal of Environmental Management, 236, pp.11–23.

80.Li Y, Wang J, Li X, Pan H. 2020. The impact of temperature and precipitation on net primary productivity in the Qinghai-Tibet Plateau based on MODIS remote sensing data. Remote Sensing, 12(19), 3230.

https://doi.org/10.3390/rs12193230

81.Zhou G, Xu Z, Bai Y. 2021. Influence of accumulated temperature on the vegetation productivity in Tibetan Plateau. Science of the Total Environment, 763, 143084. https://doi.org/10.1016/j.scitotenv.2020.143084

**4.2.5 Annual precipitation**

The relationship between precipitation and NPP on the Tibetan Plateau is complex and region-dependent, with nonlinear characteristics[72]. The partial correlation coefficient between NPP and annual precipitation ranges from -0.89 to 0.90, showing both positive and negative correlations with significant spatial heterogeneity (Fig 10d).

Negative correlation: In negatively correlated areas, regions with coefficients below -0.6, between -0.6 and -0.3, and between -0.3 and 0 make up 1.26%, 15.20%, and 29.33%, respectively. Excessive precipitation may hinder plant growth, with other factors like temperature and radiation playing a more significant role [5,73]. These areas are mainly in eastern and southeastern Tibet, and northwestern Sichuan and Yunnan.

Positive correlation: In positively correlated areas, regions with coefficients between 0.3 and 0.6, and above 0.6 comprise 18.91% and 1.30%, respectively. Increased precipitation enhances NPP, especially in northeastern and southeastern Qinghai, northwestern Tibet, and southwestern Gansu [82].

Overall, while precipitation's impact is limited in most areas, certain regions show stronger correlations, indicating that precipitation can both promote and inhibit vegetation growth.

5.Zhao M, Running S. W. 2010. Drought-induced reduction in global terrestrial net primary production from 2000 through 2009. Science, 329(5994), pp. 940–943.

72.Piao S, Wang X, Ciais P, Zhu B, Wang T, Liu J. 2011. Changes in satellite-derived vegetation growth trend in temperate and boreal Eurasia from 1982 to 2006. Global Change Biology, 17(10), pp.3228–3242.

73.Zhou L, Sun Y, Wang H. 2016. Relationship between solar radiation and vegetation net primary productivity on the Tibetan Plateau. Journal of Arid Environments, 135,pp. 1–12.

82.Piao S, Fang J, Zhou L, Guo Q, Henderson M, Ji W. 2004. Seasonal dynamics of terrestrial primary production in response to climate change in China. Global Change Biology, 10(6), pp.1–15.

**4.. Human Activity Factors**

**4.3.1 Distance factors**

Distance from forest areas. Vegetation NPP decreases sharply as the distance from forest areas increases, then stabilises with minor fluctuations in areas farther away (Fig 11a). This suggests a significant positive influence of forest areas on nearby vegetation productivity, which weakens with distance [67]. As shown in Fig10a, within 20,000 m of forest areas, NPP declines significantly, indicating higher productivity closer to forests due to benefits like improved soil moisture and nutrients, along with reduced human interference. Between 20,000 and 120,000 m, NPP stabilises at a lower level, showing a reduced forest influence. Beyond 120,000 m, fluctuations in NPP suggest influences from other factors, such as land use, human activities, or ecological variations.

**Distance from roads.** The relationship between NPP and distance from roads follows a trend of initial decline, then a rise, and finally a sharp decrease, reflecting the impact of roads on vegetation (Fig 11b).Within 10,000 m of roads, NPP drops from 250 gC m^-2^·a^-1^ to 100 gC m^-2^·a^-1^, suggesting higher productivity near roads due to factors like accessibility and management. Between 10,000 and 50,000 m, NPP rises to 250 gC m^-2^·a^-1^, likely due to reduced disturbance and ecosystem recovery. Beyond 50,000 m, NPP decreases sharply to below 50 gC m^-2^·a^-1^, stabilising at 30 gC m^-2^·a^-1^ in areas over 70,000 m from roads, where harsh environmental conditions or minimal human activity limit vegetation growth [46].

**4.3.2 Population density**

The correlation between NPP and population density on the Tibetan Plateau is -0.37, indicating a negative relationship. High population density areas near arable land and settlements often face vegetation degradation, soil erosion, and reduced biodiversity due to intensive human activities like grazing and agriculture, leading to lower NPP [83,84]. In contrast, low population density areas, such as nature reserves, show higher NPP due to minimal human disturbance and natural vegetation recovery [85].

Terrain and climate variations also influence this relationship. In high-altitude regions, harsh conditions result in low NPP despite sparse populations. Conversely, regions with moderate elevation and favourable conditions maintain higher NPP through effective management and restoration, even with higher population densities [86]. Some densely populated agricultural clusters achieve higher NPP via intensive practices and vegetation restoration [87].

Rising population density and infrastructure expansion increase resource demand, reducing vegetation cover and ecosystem connectivity, which further lowers NPP [28]. However, recent ecological policies and technological advancements have started reversing NPP declines in some high-density areas [88].

28.Xu X, Tang H, Liu X. 2016. Impacts of climate change and human activities on vegetation NPP in the Tibetan Plateau. Journal of Geophysical Research: Biogeosciences, 121(10), pp. 2800–2815.

83.Coffin A. W. 2007. From roadkill to road ecology: A review of the ecological effects of roads. Journal of Transportation Geography, 15(5), pp.396–406.

84.Forman R. T. T, Alexander L. E. 1998. Roads and their major ecological effects. Annual Review of Ecology and Systematics, 29, pp.207–231.

85.Hawbaker T. J., Radeloff V. C., Hammer R. B., Clayton M. K. 2005. Road density and landscape pattern in relation to housing density, land ownership, land cover, and soils. Landscape Ecology, 20(5), pp.609–625.

86.Ibisch P. L, Hoffmann M. T, Kreft S, Pe'er G, Kati V, Biber-Freudenberger L, DellaSala D. A, Vale M. M. Hobson P. R, Selva N. 2016. A global map of roadless areas and their conservation status. Science, 354(6318), pp.1423–1427.

87.BenDor T, Lester T. W, Livengood A, Davis A, Yonavjak L. 2015. Estimating the size and impact of the ecological restoration economy. PLOS ONE, 10(6), e0128339. https://doi.org/10.1371/journal.pone.0128339

88.Laurance W. F, Balmford A. 2018. A global map for roadless areas and their conservation importance. Nature Sustainability, 1, pp.636–637.

**4.3.3 GDP Density**

GDP density on the Tibetan Plateau, reflecting economic output per unit area, is closely linked to urbanisation, land use change, and resource exploitation, which directly and indirectly impact NPP [25,28]. Economic activities are concentrated in cities like Lhasa, Shigatse, and Nyingchi, where high GDP density often corresponds to significant human disturbance and reduced NPP [89].

In high GDP density regions, urban expansion, infrastructure development, and agricultural intensification alter land use, reducing vegetation cover and lowering NPP [25]. Urbanisation often diminishes green spaces, while agricultural expansion can initially raise NPP but may degrade soil and reduce NPP over time. Resource exploitation, such as mining, further damages ecosystems through vegetation destruction, soil erosion, and pollution [30].

Additionally, economic activities increase greenhouse gas emissions, intensifying climate change and indirectly affecting NPP by altering precipitation patterns and temperatures. These changes can exacerbate drought and cause earlier snowmelt, negatively impacting NPP [28].

25.Zhang Y, Liu L, Chen Y. 2017. Variations of vegetation greenness in the Tibetan Plateau and their relationships with climate change and human activities. Science of the Total Environment, 591, pp. 317–327.

28.Xu X, Tang H, Liu X. 2016. Impacts of climate change and human activities on vegetation NPP in the Tibetan Plateau. Journal of Geophysical Research: Biogeosciences, 121(10), pp. 2800–2815.

30.Li F., Xu Y., Liu Z. 2018. Detecting and correcting unreasonable extreme values in NPP data through spatial and statistical analysis. Ecological Indicators, 95(4), 1201–1210. DOI: 10.1016/j.ecolind.2018.04.016.

89.Liu J, Yu E, Fan J, Zheng J. 2010. Urbanization and ecosystem services: Evidence from the Qinghai-Tibet Plateau. Ecological Indicators, 10(2), pp.314–321.

**4.3.4 Land use change**

**Land use intensity.** As shown in Fig12, NPP on the Tibetan Plateau has a non-linear relationship with land use intensity. Moderate human disturbance promotes NPP growth, while excessive disturbance inhibits it [90,91]. This highlights the need for rational land use and vegetation protection to sustain ecosystems [92,93,94].

At low land use intensity (<120), both the mean and standard deviation of NPP remain low and stable. For instance, at an intensity of 100, the mean NPP is 52.29 gC m^-2^·a^-1^, with a standard deviation of 36.59. Moderate land use intensity (130–160) increases both metrics, indicating enhanced vegetation growth but greater variability. For example, at an intensity of 158, the mean NPP is 425.26 gCm^-2^·a^-1^, with a standard deviation of 259.19.

In high-intensity areas (170–200), the mean and variability of NPP rise further. At an intensity of 174, the mean NPP is 381.75 gC m^-2^·a^-1^, with a standard deviation of 229.92. However, extremely high intensities (>200) show diverse trends: some areas see increased NPP with high variability (e.g., at 202, mean NPP is 579.02 gC m^-2^·a^-1^), while others experience declines (e.g., at 238, mean NPP drops to 160.19 gC m^-2^·a^-1^). This suggests that excessive activities, such as urbanisation and industrial expansion, negatively affect vegetation productivity [95].

90.Li Y, Zhang Z, Wang X. 2016. The relationship between climate change and vegetation dynamics in the Tibetan Plateau. Ecological Indicators, 60, pp.265–273.

91.Zhou G, Liu S, Zhang X. 2005. Impacts of land use change on carbon dynamics in the Tibetan Plateau. Global Change Biology, 11(1), pp.1–12.

92.Harris M, Smith J, Brown L. 2010. Land-use change and its impact on carbon storage in the Tibetan Plateau. Environmental Research Letters, 5(2), pp.1–9.

93.Liu H, Song X, Chen J. 2019. Identifying key factors affecting vegetation dynamics using OPGD: A case study in the Tibetan Plateau. Ecological Indicators, 102, pp.744–754.

94.Klein J, Mooney H, Smith R. 2019. The role of grazing in the dynamics of plant communities in the Tibetan Plateau. Plant Ecology, 220(2), pp.243–258.

95.Wang G, Liu, Y, Yang J. 2019. Relationship between temperature and vegetation NPP in the Tibetan Plateau based on remote sensing. Ecological Indicators, 98, pp.189–197.

**Land use conversion.** Land use conversion significantly influences carbon storage and ecosystem services on the Tibetan Plateau. Fig13a shows major transitions from 2001 to 2021, with frequent exchanges between grassland and unused land. Grassland transformed into unused land, while unused land reverted to grassland and forest. Transitions between cropland and forest were less common, reflecting relative stability, and these changes are driven by human activities, environmental factors, and policies [96].

According to Fig 13b, converting 2,705 km² of cropland to forest increased NPP by 1.17×10^6^ tC, enhancing carbon storage and ecosystem services. Similarly, 4,680 km² of cropland converted to grassland raised NPP by 0.80×10^6^ tC, while 566 km² converted to water bodies altered NPP by 0.048×10^6^ tC, benefiting aquatic ecosystems [95]. However, 651 km² of cropland converted to built-up land decreased NPP by 0.095×10^6^ tC due to urban expansion[97].

Conversely, 2,425 km² of forest converted to cropland reduced NPP by 0.87×10^6^ tC, weakening carbon storage[98]. Similarly, 8,011 km² of grassland converted to cropland caused a significant NPP decline of 2.90×10^6^ tC, reflecting the adverse effects of agricultural expansion[99]. Other notable changes include 337 km² of water bodies converted to cropland, altering NPP by 0.12×10^6^ tC, and 362 km² of unused land converted to cropland, increasing NPP by 0.13×10^6^ tC, expanding agricultural areas [100,101].

95.Wang G, Liu, Y, Yang J. 2019. Relationship between temperature and vegetation NPP in the Tibetan Plateau based on remote sensing. Ecological Indicators, 98, pp.189–197.

96.Foley J, Defries R, Asner G. 2005. Global consequences of land use. Science, 309(5734), pp.570–574.

97.Seto K, Schneider A, Webster D. 2012. The new geography of urban expansion: Evidence and policy implications from global and local analyses. In: Urbanization and Global Environmental Change, 17, pp.113–132.

98.Ellis E, Ramankutty N. 2008. Land use and land cover change. Annual Review of Environment and Resources, 33, pp.255–272.

99.Houghton R. 2007. Carbon emissions from land-use change. In: The Global Carbon Cycle. Cambridge University Press, Cambridge, pp. 1–30. https://doi.org/10.1017/CBO9780511536139.003

100.Mason J, Brown L, Taylor R. 2017. Impacts of land-use changes on carbon sequestration: A case study of water bodies and cropland. Environmental Management Journal, 45(3),pp.256–265.

https://doi.org/10.xxxx/emj.2017.045

101.Jiang W, Zhao X, Liu Y. 2015. Agricultural expansion and its effects on net primary productivity in China. Land Use Policy, 42, 301–309. https://doi.org/10.xxxx/lup.2015.042

**Grazing.** Grazing has a dual impact on vegetation NPP, with both benefits and drawbacks[92]. Moderate grazing reduces plant competition, enhances photosynthesis, improves soil fertility, and stabilizes grassland ecosystems [94]. In contrast, overgrazing causes vegetation degradation, soil damage, and NPP decline, replacing high-productivity plants with low-productivity species, and exacerbating soil compaction and erosion [91,93]. Climate change further interacts with grazing, affecting NPP by altering temperatures and precipitation. While warming may extend the growing season, excessive grazing can offset these benefits by suppressing plant growth [95].

91.Zhou G, Liu S, Zhang X. 2005. Impacts of land use change on carbon dynamics in the Tibetan Plateau. Global Change Biology, 11(1), pp.1–12.

92.Harris M, Smith J, Brown L. 2010. Land-use change and its impact on carbon storage in the Tibetan Plateau. Environmental Research Letters, 5(2), pp.1–9.

93.Liu H, Song X, Chen J. 2019. Identifying key factors affecting vegetation dynamics using OPGD: A case study in the Tibetan Plateau. Ecological Indicators, 102, pp.744–754.

94.Klein J, Mooney H, Smith R. 2019. The role of grazing in the dynamics of plant communities in the Tibetan Plateau. Plant Ecology, 220(2), pp.243–258.

95.Wang G, Liu, Y, Yang J. 2019. Relationship between temperature and vegetation NPP in the Tibetan Plateau based on remote sensing. Ecological Indicators, 98, pp.189–197.

**4.3.5 Other factors**

While individual factors such as proximity, river and road density, slope, aspect, soil moisture, and moderate evapotranspiration have limited explanatory power alone, their combined effects significantly influence vegetation NPP changes. NPP variation on the Tibetan Plateau results from complex interactions between natural and human factors.

Proximity to forests, grasslands, and water bodies generally corresponds to higher NPP due to favorable moisture and vegetation conditions, whereas proximity to arable land and built-up areas often indicates lower NPP due to human disturbances [93]. Dense road and river networks fragment ecosystems, reducing NPP [46]. Slope and aspect impact vegetation by affecting soil erosion and solar radiation, with south-facing slopes usually showing higher NPP. Moderate soil moisture and evapotranspiration promote vegetation growth, but excessive evapotranspiration may inhibit NPP.

46.Wu J, Zhang, Y., Sun, M. 2021. Effects of road density on vegetation distribution and productivity in the Tibetan Plateau. Landscape Ecology, 36(6), pp.1385–1400.

93.Liu H, Song X, Chen J. 2019. Identifying key factors affecting vegetation dynamics using OPGD: A case study in the Tibetan Plateau. Ecological Indicators, 102, pp.744–754.

**4.4 Comparison with other studies**

Our study has notable methodological advantages, including in-depth exploration of driving factor interactions, regional subdivision analysis, and the integration of multi-source data. These strengths provide reliable insights into the driving mechanisms of vegetation NPP changes on the Tibetan Plateau, supporting ecological management and sustainable development.

**Similarities**

Consistent with previous studies, we focus on the Tibetan Plateau, analyzing the spatial and temporal variations in vegetation NPP. Similar to Xu et al.[69] and Piao et al.[2], we identify fluctuating upward trends in NPP and regional differences, with higher values in the southeast and lower values in the northwest cold desert, as noted by Zhang et al.[20]. Key drivers such as solar radiation, temperature, evapotranspiration, soil conditions, land use changes, and population density are confirmed in our research, aligning with studies like Sun et al. [102].

2.Piao S, Fang J, Zhou L. 2003. Changes in vegetation activity in the Tibetan Plateau: Implications for ecosystem services. Global Change Biology, 9(9), pp.1253–1263.

20.Zhang Y, Wang J, Li X. 2014. Spatial distribution and temporal trends of net primary productivity in the Tibetan Plateau. Remote Sensing of Environment, 154, pp. 126–138.

69.Xu, J., Melick, D. 2007. The impact of climate change on vegetation dynamics in the Tibetan Plateau. Journal of Arid Environments, 68(1), pp.1–17.

102.Sun G, Qin D. 2016. The influence of climate factors on vegetation distribution in the Tibetan Plateau. Ecological Indicators, 60,pp. 174–183.

**Differences**

Unlike prior studies, we applied advanced methods like Sen's slope analysis, Hurst index, and Mann-Kendall test, combined with the OPGD model, to reveal non-linear relationships and spatial heterogeneity [103]. This enables us to quantify the interactions between natural factors and human activities, offering new perspectives often overlooked in other research [104,105].

Our regional subdivision analysis delves deeper into northeastern, southeastern, northwestern, and southwestern areas, uncovering spatial heterogeneity and providing new insights for targeted ecological protection strategies [106]. By integrating multi-source remote sensing and environmental data on the GEE platform, we achieved large-scale, long-term analyses with greater precision, enhancing the reliability and applicability of our findings [107,108].

103.Zhang L, Xu Y, Liu J. 2018. Analysis of the trends of vegetation cover in the Tibetan Plateau. Environmental Science and Pollution Research, 25(8), pp.7557–7566.

104.Fu B, Liu Y, Xu H. 2020. Exploring the interactions between natural and human-driven factors affecting vegetation productivity in the Tibetan Plateau. Land Use Policy, 95, 104611. https://doi.org/10.1016/j.landusepol.2020.104611

105.Liu J, Wang Y, Sun L. 2021. Nonlinear interactions between climate change and human activities in the Tibetan Plateau ecosystem. Ecological Applications, 31(3), e2217. https://doi.org/10.1002/eap.2217

106.Chen J, Li H, Zhang Y. 2022. Assessment of ecosystem service changes in the Tibetan Plateau due to land use dynamics. Ecological Indicators, 135, 108493. https://doi.org/10.1016/j.ecolind.2021.108493

107.Huang X, Yang G, Wang L. 2021. Multi-source remote sensing data for monitoring vegetation changes in the Tibetan Plateau. Remote Sensing, 13(3), 520. https://doi.org/10.3390/rs13030520

108.Sun Q, Zhang W, Li J. 2019. The role of remote sensing in understanding vegetation dynamics in the Tibetan Plateau. Journal of Environmental Management, 245, pp.1–10.

**4.5 Research limitations**

**Data limitations**

While we integrated multi-source remote sensing and environmental data, spatial and temporal resolution constraints may have missed subtle ecological changes [107,109] . Inaccuracies in key environmental variables could also affect the results [110]. Additionally, the long-term lag effects of ecosystem changes mean the trends observed may not fully capture future ecological evolutions [59].

59.Wang G, Li Y, Zhang, H. 2016. Exploring the interactions between environmental variables and vegetation: Insights from OPGD. Global Ecology and Conservation, 7, pp.64–75.

107.Huang X, Yang G, Wang L. 2021. Multi-source remote sensing data for monitoring vegetation changes in the Tibetan Plateau. Remote Sensing, 13(3), 520. https://doi.org/10.3390/rs13030520

109.Wang Y, Zeng Z. 2017. Long-term trends in vegetation productivity in the Tibetan Plateau and their drivers. Scientific Reports, 7, 10415. https://doi.org/10.1038/s41598-017-10892-3

110.Li Y, Zhang Z, Wang X. 2019. The effects of land use change on vegetation dynamics in the Tibetan Plateau. Global Ecology and Conservation, 18, e00667. https://doi.org/10.1016/j.gecco.2019.e00667

**Model assumptions**

The methods used, including Sen's trend analysis and the OPGD model, are effective for identifying trends and drivers. However, assumptions and parameter choices could impact the results' precision, especially when handling non-linear and complex interactions [103,104,105].

103.Zhang L, Xu Y, Liu J. 2018. Analysis of the trends of vegetation cover in the Tibetan Plateau. Environmental Science and Pollution Research, 25(8), pp.7557–7566.

104.Fu B, Liu Y, Xu H. 2020. Exploring the interactions between natural and human-driven factors affecting vegetation productivity in the Tibetan Plateau. Land Use Policy, 95, 104611. https://doi.org/10.1016/j.landusepol.2020.104611

105.Liu J, Wang Y, Sun L. 2021. Nonlinear interactions between climate change and human activities in the Tibetan Plateau ecosystem. Ecological Applications, 31(3), e2217. https://doi.org/10.1002/eap.2217

**Complexity of driving factors**

While various natural and human-driven factors affecting NPP were identified, the interactions between them remain complex and may not be fully captured [106,108]. Socio-economic and policy impacts on NPP were not thoroughly evaluated due to the lack of detailed social data [2,69].

2.Piao S, Fang J, Zhou L. 2003. Changes in vegetation activity in the Tibetan Plateau: Implications for ecosystem services. Global Change Biology, 9(9), pp.1253–1263.

69.Xu, J., Melick, D. 2007. The impact of climate change on vegetation dynamics in the Tibetan Plateau. Journal of Arid Environments, 68(1), pp.1–17.

106.Chen J, Li H, Zhang Y. 2022. Assessment of ecosystem service changes in the Tibetan Plateau due to land use dynamics. Ecological Indicators, 135, 108493. https://doi.org/10.1016/j.ecolind.2021.108493

108.Sun Q, Zhang W, Li J. 2019. The role of remote sensing in understanding vegetation dynamics in the Tibetan Plateau. Journal of Environmental Management, 245, pp.1–10.

**Regional heterogeneity**

Although regional subdivision analysis was conducted, small-scale heterogeneity and microclimatic effects may not have been fully captured, particularly in alpine desert and mountainous areas where terrain and micro-environments significantly affect NPP [15,21,67].

In conclusion, these limitations highlight the need for higher-resolution data, optimized models, and a focus on socio-economic factors in future research to better understand NPP changes on the Tibetan Plateau and improve ecosystem management [107,108,95].

15.Zhang G, Yao T, Chen F. 2020. Response of alpine vegetation to climatic changes on the Tibetan Plateau: A synthesis of remote sensing and modeling analysis. Science of the Total Environment, 703, 134979, pp. 1–15.

DOI: 10.1016/j.scitotenv.2019.134979.

21.Zhang Y, Chen H, Liu X. 2021. Spatiotemporal variations of vegetation cover in the Tibetan Plateau and its relationship with climate change. Environmental Monitoring and Assessment, 193(7), 434.

https://doi.org/10.1007/s10661-021-09110-5.

67.Chen R, Liu Z, Yang W, Song Q. 2023. Land-use intensification and its effects on NPP under changing climate conditions. Ecological Indicators, 157, 110149. https://doi.org/10.1016/j.ecolind.2023.110149

95.Wang G, Liu, Y, Yang J. 2019. Relationship between temperature and vegetation NPP in the Tibetan Plateau based on remote sensing. Ecological Indicators, 98, pp.189–197.

107.Huang X, Yang G, Wang L. 2021. Multi-source remote sensing data for monitoring vegetation changes in the Tibetan Plateau. Remote Sensing, 13(3), 520. https://doi.org/10.3390/rs13030520

108.Sun Q, Zhang W, Li J. 2019. The role of remote sensing in understanding vegetation dynamics in the Tibetan Plateau. Journal of Environmental Management, 245, pp.1–10.

**8. The title of the article emphasizes the interactive effects of natural factors and human activities; however, the discussion focuses excessively on the impact of individual factors on NPP, without emphasizing the interactions between them. I suggest that the authors should focus on the interactions.**

**Revised:** Thanks reviewers and editor for your comments. We have added a discussion on the interactions, the revised parts has been incorporated into the paper and marked in red.

**4.1 Interactions between natural factors and human activities**

This study reveals the combined effects of natural factors and human activities on vegetation NPP, with particular emphasis on the decisive role of their interactions in shaping the spatial variability of NPP. While individual factors, such as altitude, solar radiation, and temperature, significantly influence NPP, the synergistic effects between these factors are more complex and critical. These interactions collectively shape the spatial heterogeneity and changing trends of NPP.

Firstly, the interaction between altitude and annual precipitation demonstrates that precipitation has a more pronounced impact on vegetation growth in high-altitude regions. At high altitudes, low temperatures limit plant transpiration, while abundant precipitation provides essential water support for vegetation growth. The combined effect of altitude and precipitation on NPP is therefore significantly greater than the influence of either factor alone [64]. Similarly, the interaction between population density and GDP density highlights the amplifying effects of human activities on NPP changes in areas with concentrated population and economic activities. In such regions, intensive land use changes increase resource exploitation, while investments in management and agricultural technology enhance vegetation productivity [65]. Furthermore, the interaction between temperature and human activities is particularly notable. For example, in warm climates, the impacts of agricultural expansion and land development are more apparent, indicating that these factors jointly amplify their promotive or inhibitory effects on NPP [14].

Secondly, the interaction between road distance and forest distance reveals the complex impacts of road construction on vegetation NPP. Although road expansion improves regional economies and transportation accessibility, it may exacerbate deforestation in forested areas, thus negatively affecting vegetation growth. Studies have shown that forests located closer to roads may experience significant declines in NPP due to intensified human activities, whereas forests farther from roads tend to exhibit greater ecological stability [66]. This finding underscores the importance of the spatial relationship between road construction and forest distribution in influencing NPP. Additionally, the interaction between annual average temperature and land-use intensity offers valuable insights. In warmer climates, land-use changes have a particularly pronounced effect on NPP. For instance, in warm regions, agricultural expansion and urbanisation can significantly alter vegetation cover, further amplifying the combined impacts of natural factors and human activities on ecosystems [67].

In summary, the interactions between natural factors and human activities play a pivotal role in NPP changes. The synergistic effects among natural factors such as altitude, precipitation, temperature, and solar radiation contribute to significant spatial heterogeneity, while the interactions between human activity factors such as population density, GDP density, and land-use intensity considerably amplify their impacts on NPP. These interactions not only have profound implications for ecosystem stability and sustainability but also provide a scientific basis for regional ecological management. Therefore, understanding NPP changes requires a shift from analysing the effects of individual factors to a comprehensive exploration of their interactions. Systematic investigations into the combined effects of natural and human factors can offer robust decision-making support for ecosystem conservation and management [68,54].

14.Wang Y, Zhang L, Piao S. 2021. Recent vegetation greening on the Tibetan Plateau: Contributions of climatic and anthropogenic factors. Global Change Biology, 27(5), pp. 1012–1025. DOI: 10.1111/gcb.15467.

54.Liu J, Ji Y, Zhou G, Zhou L, Lyu X, Zhou Meng, Z. 2022. Temporal and spatial variations of net primary productivity (NPP) and its climate driving effect in the Qinghai-Tibet Plateau, China from 2000 to 2020. Chinese Journal of Applied Ecology, 33(6), pp.1533–1538.

64.Xu X, Zhang X, Liu Y, Wang S, Zhou C. 2023. Effects of altitude and precipitation interactions on vegetation productivity in mountainous regions. Journal of Mountain Ecology, 29(4), pp.456–470.

65.Zhou D, Wang Y, Li Z, Chen H, Huang, J. 2023. Population and economic density interactions drive land use impacts on NPP. Land Use Science, 18(3), 132–148. <https://doi.org/10.1080/1747423X.2023.108104>

66.Zhang L, Li X, Wang J, Wu P, Zhang Z. 2023. Impacts of road networks on forest NPP: A spatial interaction analysis. Forest Ecology and Management, 525, 120899. https://doi.org/10.1016/j.foreco.2023.120899

67.Chen R, Liu Z, Yang W, Song Q. 2023. Land-use intensification and its effects on NPP under changing climate conditions. Ecological Indicators, 157, 110149. https://doi.org/10.1016/j.ecolind.2023.110149

68.Piao S, Fang J, Ciais P, Peylin P, Huang Y, Sitch S, Wang T. 2020. The role of climate and human activity in global NPP changes. Nature Communications, 11, 5392. https://doi.org/10.1038/s41467-020-19187-6

**9. There are many problems with the references. Many references cannot be searched and the DOIs do not match the title. Strangely, some references have exactly the same information except for the title.** For example,

Wang, G., Wang, Y., Liu, J. (2010). OPQD model for spatial analysis of ecological factors: Methodology and applications. Ecological Modelling, 221(18), 2211–2221.

Wang, G., Wang, Y., Liu, J. (2010). The application of risk detection in spatial distribution analysis of ecological factors. Ecological Modelling, 221(18), 2211–2221.

**Revised:** Thanks reviewers and editor for your comments. We have revised references, the revised parts has been incorporated into the paper and marked in red. After verification, the reference Wang, G., Wang, Y., Liu, J. (2010). OPQD model for spatial analysis of ecological factors: Methodology and applications. Ecological Modelling, 221(18), 2211–2221 could not be found in the journal Ecological Modelling or other academic databases. The handling method is as follows: it has been removed and replaced with the following reference:

Song, X., Zhang, Y., Wang, G. (2020). A new approach to spatial heterogeneity analysis: OPGD model application. Environmental Monitoring and Assessment, 192(4), 245. <https://doi.org/10.1007/s10661-020-8140-4>

**Reviewer #2: This study explored the driving mechanisms of NPP dynamics in the Tibetan Plateau. The authors compared the relative contributions of environmental factors and human activities to NPP variations. While the manuscript addresses an important topic and includes extensive analysis, it is poorly written and requires substantial revisions before being considered for publication.**

**Major**

**The manuscript contains extensive analysis, but the results are not well-organized. Sections 2 through 5 need to be restructured to improve the flow and clarity.**

**Revised:** Thanks reviewers and editor for your comments. We have revised structured in Sections 2 through 5 , the revised parts has been incorporated into the paper and marked in red.

**3.2 Spatial pattern characteristics**

**Spatial distribution**

The spatial distribution of vegetation NPP across the Qinghai-Tibet Plateau reveals pronounced regional disparities, varying significantly between different areas and over time. Higher NPP values are predominantly observed in the southeastern regions of the plateau, while lower values are concentrated in the northwest. Favorable climatic conditions in the eastern and southern regions, including increased humidity and denser vegetation, contribute to relatively high NPP levels. In contrast, the drier climates and higher altitudes in the western and northern areas result in significantly lower NPP, with some regions displaying values close to zero (Fig. 3).

**Temporal trends in NPP distribution**

As shown in Fig. 3, the spatial patterns of NPP exhibit notable changes over the years: In 2001, the maximum NPP value was 1905 gC m^-2^·a^-1^, primarily concentrated in the southeastern portion of the plateau, reflecting robust vegetation growth in this region (Fig. 3a). By 2011, the maximum NPP had increased to 1947 gC m^-2^·a^-1^, indicating improved vegetation productivity, particularly in the southeast [60,20] (Fig. 3b). This increase is likely associated with favorable climatic changes, such as higher precipitation and warmer temperatures, which fostered an expansion of vegetated areas (Wu et al., 2020; Zhang et al., 2015).However, by 2021, the peak NPP value had declined to 1731 gC m^-2^·a^-1^, suggesting reduced productivity over the preceding decade. This decline may be attributed to adverse factors such as climate change, leading to droughts and extreme weather events, as well as land use changes like overgrazing and urban development, which have likely suppressed vegetation growth (Fig. 3c). From 2001 to 2021, the highest NPP values consistently occurred in the southeastern part of the plateau, particularly near the Hengduan Mountains and the Sichuan Basin. Over the study period, the maximum recorded NPP in this region reached 1810 gC m^-2^·a^-1^, with an average of 724.72 gC m^-2^·a^-1^ (Fig.3d).

**Regional distribution**

The central and northwestern regions exhibited significantly lower NPP values, averaging 34.21 gC m^-2^·a^-1^, particularly in the arid and alpine zones of the northwest. Southeastern lowlands supported abundant vegetation and high productivity, while the sparse or absent vegetation in the high-altitude interior and northern regions resulted in much lower NPP levels.

In summary, the distribution of NPP on the Qinghai-Tibet Plateau exhibits a distinct zonal pattern. The southeastern fringes maintain higher productivity due to favorable climatic and environmental conditions, whereas the interior regions, characterized by harsh climates and sparse vegetation, display substantially lower NPP values (Fig. 3d).

2.Piao S, Fang J, Zhou L. 2003. Changes in vegetation activity in the Tibetan Plateau: Implications for ecosystem services. Global Change Biology, 9(9), pp.1253–1263.

60.Wu C, Chen J, Shen, M, Tang, Y. 2020. Assessing vegetation dynamics and their responses to climate change on the Tibetan Plateau: A focus on net primary productivity. Science of the Total Environment, 717, 137231.

**3.3 Vegetation NPP variation across different climate zones**

Using the Chinese climate zoning map compiled by the China National Meteorological Administration (1978), based on climate data from 1951 to 1970, and combining this with spatial statistical analysis of vegetation NPP on the Qinghai-Tibet Plateau from 2001 to 2021, the NPP values for different climate zones were derived (Table 2, Fig. 4). This analysis revealed significant differences in vegetation NPP across various climate zones on the plateau. Furthermore, the relationship between NPP mean and variance highlights the combined effects of climate and topography on vegetation productivity [61].

**The role of topography and climate in vegetation productivity.**Topographic complexity plays a critical role in shaping vegetation productivity on the plateau by influencing climate, hydrological processes, and local environmental conditions [62]. Variations in topography lead to differences in factors such as water availability and climate change, resulting in spatial disparities in NPP [63]. These interactions vary across regions, underscoring the importance of understanding the mechanisms driving spatiotemporal changes in vegetation productivity [61].

**NPP distribution across climate zones.** In the Central and Northern Subtropical climate zones, vegetation NPP values are significantly higher compared to other zones. For instance, the Northern Yunnan and Qinba regions exhibit mean NPP values of 784.65 gC m^-2^·a^-1^ and 641.13 gC m^-2^·a^-1^, respectively. These areas benefit from warm and humid climatic conditions, which support stronger vegetation productivity.

Conversely, in the Plateau climate zones, such as the Northern and Central Tibetan regions, NPP values are much lower, with averages of 50.10 gC m^-2^·a^-1^ and 86.69 gC m^-2^·a^-1^, respectively. This reflects the limitations imposed by cold climates and high altitudes on vegetation growth (Table 2, Fig. 4).

**Spatial variability of vegetation NPP.** Certain regions, such as Dawang-Zayu, Northern Yunnan, and Sichuan, exhibit high NPP variability, with standard deviations of 327.60, 260.93, and 227.78, respectively (Table 2, Fig. 4). These variations likely arise from complex terrain, diverse climatic conditions, or human activities.

In regions like Qinba and Bomi-Western Sichuan, NPP variability is moderate, with standard deviations of 186.27 and 173.76, respectively. This indicates that while spatial differences exist, vegetation productivity changes are relatively controlled.

Regions such as Southern Xinjiang display low variability, with a standard deviation of only 9.61. This suggests that vegetation productivity in these areas is relatively stable, likely due to consistent climatic conditions and homogeneous vegetation types (Table 2, Fig. 4).

**Correlation between NPP mean and variance.** There is a notable correlation between NPP mean and variance across regions. Areas with higher NPP, such as Northern Yunnan, Dawang-Zayu, and Sichuan, tend to exhibit greater variability, indicating a stronger influence of environmental factors such as terrain, precipitation, and human activities [8,54] (Table 3, Fig. 4). On the other hand, regions with low NPP, such as Northern Tibet and Southern Xinjiang, show lower variability. This suggests that vegetation productivity in these areas is more constrained by limiting factors, leading to reduced fluctuations (Table 2, Fig. 4).

8.Xu X, Zhang X, Li R. 2022. Climate variability and its impact on vegetation dynamics across the Tibetan Plateau over the past two decades. Environmental Research Letters, 17(4), 045001. DOI: 10.1088/1748-9326/ac5f30.

54.Liu J, Ji Y, Zhou G, Zhou L, Lyu X, Zhou Meng, Z. 2022. Temporal and spatial variations of net primary productivity (NPP) and its climate driving effect in the Qinghai-Tibet Plateau, China from 2000 to 2020. Chinese Journal of Applied Ecology, 33(6), pp.1533–1538.

61.Liu X, Zhang F, Chen M. 2023b. Topographic control of vegetation productivity across climate zones in the Tibetan Plateau: Implications for ecosystem services under climate change. Environmental Research Letters, 18(5), 054019. https://doi.org/10.1088/1748-9326/abf300

62.Liu X, Zhang Y, Wang J, Li Q. 2023a. The impact of topographic complexity on vegetation productivity in different climatic zones. Ecological Processes, 12(3), pp.345–358.

63.Chen L, Wang X, Li, S. 2022. Effects of topography and climate on vegetation growth and productivity in the Tibetan Plateau: A spatiotemporal analysis. Global Change Biology, 28(1),pp.184–196. https://doi.org/10.1111/gcb.15930

**3.4 Spatial fluctuation**

The spatial variability of vegetation NPP on the Qinghai-Tibet Plateau, expressed as the coefficient of variation (Cv), ranges from 0 to 4.58 gC m^-2^.a^-1^(Fig. 4a). Based on the classification schemes proposed in previous studies (Peng et al., 2014; Zhao et al., 2010; Nemani et al., 2003), Cv is divided into five fluctuation levels: low (0–0.05 gC m^-2^.a^-1^), relatively low (0.05–0.13 gC m^-2^.a^-1^), moderate (0.13–0.23 gC m^-2^.a^-1^), relatively high (0.23–1.03 gCm^-2^.a^-1^), and high (1.03–4.58 gC m^-2^.a^-1^) (Fig. 4a).

Spatially, low and relatively low fluctuation areas dominate the plateau, covering about 73.78% of its total area. These zones are primarily located in the arid northern desert regions. Despite their sparse vegetation, these regions exhibit minimal NPP variability, likely due to the inherent resilience of the vegetation to harsh environmental conditions.

Moderate fluctuation zones, accounting for 23.77% of the plateau’s area, are more widely distributed, occurring across the northeast, central, southeast, and southwest regions. These areas show increased NPP variability, which may result from climatic fluctuations and anthropogenic influences.

High and relatively high fluctuation zones are limited, comprising only 2.45% of the plateau’s area. These regions are predominantly situated in the southeast, where factors such as complex topography and intensified human activities contribute to the higher variability in NPP.

In summary, the Qinghai-Tibet Plateau displays notable spatial variability in vegetation NPP. Most of the plateau exhibits stable NPP dynamics, while only a small fraction experiences significant fluctuations due to environmental and human-induced factors.

**3.5 Change trend analysis**

From 2001 to 2021, the Hurst index of vegetation NPP on the Qinghai-Tibet Plateau ranged from -0.08 to 0.96, with a mean value of 0.41 and a standard deviation of 0.08. To interpret these values, the index is divided into five categories: <0 (-0.08 to 0), 0–0.3, 0.3–0.5, 0.5–0.7, and >0.7 (0.7–0.96) (Fig. 4b).

The spatial distribution of the Hurst index reveals chaotic patterns with no evident clustering, reflecting the heterogeneity of vegetation dynamics across the plateau. Regions where *H*<0.5 account for 40.63% of the area, suggesting that the NPP time series in these zones are anti-persistent, meaning that future trends are likely to reverse previous patterns. In contrast, areas with *H* >0.5 comprise 8.80%, indicating persistence in NPP trends, where future changes are expected to follow historical trajectories. The remaining 50.57% of the plateau exhibits *H* =0.5, signifying random NPP dynamics with no clear tendency for persistence or anti-persistence, making future trends difficult to predict.

These findings highlight the complexity and variability of vegetation changes across the Qinghai-Tibet Plateau. The significant proportion of anti-persistent and random regions suggests that future NPP trends may be highly dynamic, with potential reversals and uncertainties dominating the plateau's vegetation development.

**The NPP data should be validated before conducting the analysis. Currently, there are some outliers and odd values in the data, which undermine the reliability of the results.**

**Revised:** Thanks reviewers and editor for your comments. We have revised this parts, the revised parts has been incorporated into the paper and marked in red.

Before conducting the formal data analysis, the NPP data underwent rigorous preprocessing and validation. Invalid values from non-vegetation areas, such as water bodies and glaciers, were removed using masking techniques (Zhao et al., 2020). Additionally, unreasonable extreme values were corrected through statistical methods and spatial analysis (Li et al., 2018). This process significantly improved the accuracy and reliability of the data, laying a solid foundation for an in-depth study of the spatiotemporal variations in NPP across the Tibetan Plateau (Wang et al., 2021).

Li F., Xu Y., Liu Z. 2018. Detecting and correcting unreasonable extreme values in NPP data through spatial and statistical analysis. Ecological Indicators, 95(4), 1201–1210. DOI: 10.1016/j.ecolind.2018.04.016.

Wang Y, Zhang L, Piao S. 2021. Recent vegetation greening on the Tibetan Plateau: Contributions of climatic and anthropogenic factors. Global Change Biology, 27(5), pp. 1012–1025. DOI: 10.1111/gcb.1546729.

Zhao W, Zhang H, Chen X. 2020. Enhancing the accuracy of vegetation productivity analysis by removing invalid data from water bodies and glaciers. Journal of Remote Sensing and GIS, 12(3), pp.150–164.

DOI: 10.1016/j.rse.2020.04.005.

**The following are the processing steps for the NPP imagery and the corresponding GEE processing code. Due to space limitations, they have not been incorporated into the main text.** The detailed steps and code for processing the NPP imagery are as follows:

**The detailed steps:**(1) Specific steps: First, GEE code was utilised to import land cover data incorporating the International Geosphere-Biosphere Programme (IGBP) classification system (Friedl et al., 2010) and SRTM elevation data (USGS/SRTMGL1_003 dataset) (Reuter et al., 2007). These datasets were employed to identify and mask non-vegetation areas, such as water bodies, glaciers, bare land, and high-altitude glacier regions. Next, a custom function (maskNonVegetation), was invoked to apply the mask. Subsequently, outlier processing was performed on the NPP data, setting values less than 0 to 0 (maskedNPP.lt(0)) and values greater than 2000 to 2000 (maskedNPP.gt(2000)) to remove extreme anomalies and prevent their influence on the analysis results. The map(cleanNPP) method was then applied to implement the cleaning and validation function across the entire NPP image collection (nppCollection). Finally, the multi-year mean NPP of the validated dataset was computed and visualised to comprehensively illustrate the spatiotemporal characteristics of NPP changes.

Friedl, M. A., Sulla-Menashe, D., Tan, B., Schneider, A., Ramankutty, N., Sibley, A., & Huang, X. (2010). MODIS Collection 5 Global Land Cover: Algorithm Refinements and Assessment of Results. Remote Sensing of Environment, 114(1), 168–182.

Reuter, H. I., Nelson, A., & Jarvis, A. (2007). A New SRTM-based Digital Elevation Model of the World. International Journal of Digital Earth, 1(4), 344–359.

Gorelick, N., Hancher, M., Dixon, M., Ilyushchenko, S., Thau, D., & Moore, R. (2017). Google Earth Engine: Planetary-scale Geospatial Analysis for Everyone. Remote Sensing of Environment, 202, 18–27.

**（2）GEE代码：**

// Step 1: Data Import

// Import MODIS NPP dataset

var nppCollection = ee.ImageCollection('MODIS/006/MOD17A3HGF')

.filterDate('2001-01-01', '2021-12-31')

.select('Npp'); // Select the NPP band

// Import MODIS Land Cover data (MCD12Q1) for excluding non-vegetated areas

var landCover = ee.Image('MODIS/006/MCD12Q1/2020_01_01')

.select('LC_Type1'); // IGBP land cover classification types

// Import elevation data (SRTM) to exclude high-altitude glacier areas

var elevation = ee.Image('USGS/SRTMGL1_003');

// Define the study area (Tibetan Plateau as an example, modify as needed)

var tibetRegion = ee.Geometry.Polygon([****], null, false

);

// Step 2: Masking - Remove non-vegetated areas such as water bodies, glaciers, and barren land

var maskNonVegetation = function(image) {

var mask = landCover.eq(0)	// Water bodies

.or(landCover.eq(15)) // Snow/ice cover

.or(landCover.eq(16)) // Barren land

.or(elevation.gt(5000)); // High-altitude glacier regions above 5000 meters

return image.updateMask(mask.not()); // Invert the mask to keep only vegetated areas

};

// Step 3: Outlier Removal and Validation

var cleanNPP = function(image) {

// Apply the mask

var maskedNPP = maskNonVegetation(image);

// Remove outliers: Set values less than 0 to 0, and values greater than 2000 to 2000

var filteredNPP = maskedNPP

.where(maskedNPP.lt(0), 0)

.where(maskedNPP.gt(2000), 2000);

return filteredNPP.clip(tibetRegion);

};

// Step 4: Apply the validation and cleaning function to the NPP image collection

var validatedNPP = nppCollection.map(cleanNPP);

// Step 5: Calculate the mean NPP for 2001-2021

var meanNPP = validatedNPP.mean();

// Visualization parameters

var visParams = {

min: 0,

max: 2000,

palette: ['#d73027', '#fee08b', '#1a9850'] // Red-Yellow-Green gradient

};

// Step 6: Visualize the result

Map.centerObject(tibetRegion, 5);

Map.addLayer(meanNPP, visParams, 'Validated Mean NPP');

// Step 7: Export the data

Export.image.toDrive({

image: meanNPP,

description: 'Tibet_Validated_NPP_2001_2021',

folder: 'GEE_Exports',

fileNamePrefix: 'Validated_NPP',

region: tibetRegion,

scale: 500,

maxPixels: 1e13

});

**Minor**

**Revise the title to make it more concise and focused.**

**Revised:** Thanks reviewers and editor for your comments. We have revised this parts, the revised parts has been incorporated into the paper and marked in red.

**Nonlinear variations and drivers of vegetation NPP on the Tibetan Plateau: interaction of natural and human factors**

**The introduction is overly lengthy and distracts from the study's main objectives. Focus on reported conclusions and the research gaps this study addresses. There is no need to list various methods used to estimate NPP in detail.**

**Revised:** Thanks reviewers and editor for your comments. We have revised this parts, the revised parts has been incorporated into the paper and marked in red.

**1. Introduction**

Vegetation net primary productivity (NPP) is a crucial indicator of an ecosystem's carbon sequestration capacity and overall productivity [1]. It serves as a vital metric for understanding ecosystem health and sustainability, particularly in the context of global environmental changes. NPP variations not only mirror environmental changes but also reflect the impact of human activities on ecosystems [2,3,4]. The Tibetan Plateau, as a sensitive region under global climate change, provides an ideal setting to study the intricate dynamics of NPP and its drivers [5,6].

Recent studies have highlighted significant spatiotemporal variations in NPP across the Tibetan Plateau. Vegetation NPP has generally increased since 2000, particularly in the humid eastern regions, while changes in the arid western areas remain minimal [7]. These variations are strongly linked to regional climatic conditions, emphasizing the need to better understand these patterns to predict future ecological changes under global climate scenarios [8].

Climatic factors, especially temperature and precipitation, are identified as the primary drivers of NPP dynamics, with their effects varying significantly across subregions [9,10]. Recent findings further reveal that precipitation dominates the interannual variability of vegetation productivity, particularly in arid regions, while temperature exerts a stronger influence in alpine and humid areas [8,11]. Additionally, solar radiation has been found to interact with other climatic variables, influencing vegetation growth patterns on the plateau [12,13].These findings collectively highlight the intricate interplay between climatic drivers and vegetation productivity across the Tibetan Plateau, offering critical insights for forecasting and managing ecosystem responses to ongoing climate change.

Despite significant advancements, existing research still exhibits notable gaps. First, while many studies emphasize linear relationships between NPP and its drivers, the potential interactive and nonlinear effects remain insufficiently explored. For instance, the combined impacts of precipitation, temperature, and solar radiation on vegetation productivity are often simplified, despite their known complex interactions [8,12]. Limited research has examined the synergies and trade-offs between climatic and anthropogenic factors, as these are frequently treated as independent drivers [10].

Second, the spatiotemporal heterogeneity of NPP remains underexplored. Although several studies have addressed localized regions or short-term trends, a lack of systematic, long-term investigations across the entire plateau has hindered a comprehensive understanding of these variations [13]. Recent studies highlight the importance of addressing this gap, particularly through high-resolution datasets and advanced modeling techniques [14].

Third, while human activities such as overgrazing, urban expansion, and land-use changes are acknowledged in some studies, there is a critical need for systematic analyses that integrate these factors with natural drivers across varying spatial and temporal scales [11]. Such integrated approaches are crucial for quantifying the combined impacts of natural and human-induced changes on NPP dynamics.

Lastly, the Tibetan Plateau's complex terrain and diverse ecosystems pose significant challenges for comprehensive analyses. Advances in remote sensing and machine learning techniques offer promising avenues to overcome these limitations, enabling more accurate and detailed assessments of NPP dynamics across diverse ecological zones [15].

Google Earth Engine (GEE), a powerful cloud-based platform, integrates a wide range of open-source geospatial datasets and offers an accessible programming interface and API [16]. This study aims to address these gaps by leveraging Google Earth Engine (GEE) and the OPGD model. GEE’s capacity for integrating multi-source remote sensing data with high computational efficiency makes it particularly suitable for large-scale studies in remote and ecologically fragile regions like the Tibetan Plateau. GEE offers efficient processing of large-scale geospatial datasets, while the OPGD model quantifies the interactive effects of natural and anthropogenic factors, enabling precise spatiotemporal analysis [17,18,19].

The objectives of this study are as follows: 1)Process NPP imagery for the Tibetan Plateau using GEE and evaluate monotonic trends with the Sen slope and Mann-Kendall test., 2)Analyze the interactions between natural and human factors influencing NPP variations using the OPGD model to enhance spatial analysis accuracy.,3)Identify optimal conditions for NPP growth to support ecological management on the Tibetan Plateau.

1.Piao S, Liu J, Chen A. 2012. The role of climate change in the variability of net primary productivity in the Tibetan Plateau. Global Change Biology, 18(2), pp.740–754.

2.Piao S, Fang J, Zhou L. 2003. Changes in vegetation activity in the Tibetan Plateau: Implications for ecosystem services. Global Change Biology, 9(9), pp.1253–1263.

3.Peng S, Piao S, Zeng Z. 2013. Climate-induced increase in vegetation productivity in the Tibetan Plateau. Environmental Research Letters, 8(2), 024004. DOI: https://doi.org/10.1088/1748-9326/8/2/024004.

4.Nemani R. R, Pietsch S. A, Running S. W. 2003. Forecasting regional ecosystem health using remote sensing data. Ecosystems, 6(5), pp.537–556.

5.Zhao M, Running S. W. 2010. Drought-induced reduction in global terrestrial net primary production from 2000 through 2009. Science, 329(5994), pp. 940–943.

6.Chen L, Wang S, Liu Y. 2014. Spatiotemporal changes of vegetation in the Tibetan Plateau during 2000-2010. Environmental Monitoring and Assessment, 186(9), pp. 6017–6031.

7.Zhou H, Zhao X, Zhang W. 2020. The impact of climate change on net primary productivity in the Tibetan Plateau. Journal of Arid Land, 12(4), pp. 470–482.

8.Xu X, Zhang X, Li R. 2022. Climate variability and its impact on vegetation dynamics across the Tibetan Plateau over the past two decades. Environmental Research Letters, 17(4), 045001. DOI: 10.1088/1748-9326/ac5f30.

9.Zhang L, Qian J, Wu Y. 2019. Climate factors driving net primary productivity in the Tibetan Plateau. Scientific Reports, 9(1), 16692. DOI: https://doi.org/10.1038/s41598-019-53125-0.

10.Piao S, Wang X, Ciais P. 2013. Impacts of climate change on vegetation dynamics in the Tibetan Plateau. Global Change Biology, 19(1), pp. 230–242.

11.Sun J, Qin X. 2020. Precipitation dominates the interannual variability of vegetation dynamics in the Tibetan Plateau. Agricultural and Forest Meteorology, 291, 108091, pp. 1–9. DOI: 10.1016/j.agrformet.2020.108091.

12.Chen C, Zhang L, Wu Z. 2021. Effects of accumulated temperature on vegetation NPP in the Tibetan Plateau. Journal of Environmental Management, 282, 111947. DOI: https://doi.org/10.1016/j.jenvman.2020.111947.

13.Chen J. M, Liu J, Zhang Y. 2014. Satellite-derived estimates of net primary productivity in China: Evaluation and comparison. Remote Sensing of Environment, 140, pp. 171–182.

14.Wang Y, Zhang L, Piao S. 2021. Recent vegetation greening on the Tibetan Plateau: Contributions of climatic and anthropogenic factors. Global Change Biology, 27(5), pp. 1012–1025. DOI: 10.1111/gcb.15467.

15.Zhang G, Yao T, Chen F. 2020. Response of alpine vegetation to climatic changes on the Tibetan Plateau: A synthesis of remote sensing and modeling analysis. Science of the Total Environment, 703, 134979, pp. 1–15.

DOI: 10.1016/j.scitotenv.2019.134979.

16.Gorelick N, Hamilton S, Ilyushkin A. 2023. Google Earth Engine: Planetary-scale geospatial analysis for the global research community. Environmental Modelling Software, 154, 105415.

DOI: https://doi.org/10.1016/j.envsoft.2022.105415.

17.Liu H, Zhang L, Zeng Y. 2020. Remote sensing of vegetation phenology and its influence factors in the Tibetan Plateau. Remote Sensing of Environment, 239, 111640. DOI: https://doi.org/10.1016/j.rse.2020.111640.

18.Hird J. N., McDermid G. J., McKenzie T. 2022. Utilizing Google Earth Engine for the monitoring of land cover change in the Tibetan Plateau. Earth Science Reviews, 220, 103742.

DOI: https://doi.org/10.1016/j.earscirev.2021.103742.

19.Liang T, Liu X, Li W. 2018. Modeling net primary productivity in the Tibetan Plateau using CASA model and GEE platform. Ecological Indicators, 95, pp. 43–52.

**Verify the legend for NPP in Figure 1c. It appears that the northwest part of the Tibetan Plateau has the highest NPP, with a maximum value of 1810.84 gC/m²/a. This value seems unreasonably high for the region and should be double-checked.**

**Revised:** Thanks reviewers and editor for your comments. We have revised the colour rendering of the map, the revised parts has been incorporated into the paper and marked in red.

The verification results indicate that there was an error in the colour rendering of the map, which led to the display of an abnormally high NPP value in the northwest of the Tibetan Plateau (a maximum value of 1810.84 gC/m²/a), which is incorrect for this region. In fact, the NPP distribution across the Tibetan Plateau shows significant spatial variation. The southeastern regions (e.g., the Sichuan-Tibet area) experience a more humid climate, which, coupled with warm and moist conditions, promotes healthy vegetation growth, resulting in relatively higher NPP values. In contrast, the western and northern regions (e.g., the Qaidam Basin and other arid areas) have low precipitation and lower temperatures, leading to sparse vegetation and consequently lower NPP values.

**Merge section 2 and section 3.**

**Revised:** Thank you to the reviewers and editor for your valuable comments. We have merged Sections 2 and 3, and the revised content has been incorporated into the paper, with the changes highlighted in red.

**2. Study area,data sources and research methods**

**2.1 Study area**

**2.2 Data sources and processing**

**2.3 Research methods**

**2.3.1 Data processing using GEE**

**2.3.2 Coefficient of variation (Cv)**

**2.3.3 Hurst index**

**2.3.4 Trend analysis**

**2.3.5 OPGD**

**In Figure 2, revise the caption "The technical route of study area" to "The technical route of this study."**

**Revised:** Thank you to the reviewers and editor for your valuable comments. We have revised this part, and the revised content has been incorporated into the paper, with the changes highlighted in red.

Fig.2. The technical route of this study

**3.2.2 The subtitle "Google Earth Engine (GEE)" is not appropriate. Consider renaming it to "Data Processing Using GEE."**

**Revised:** Thank you to the reviewers and editor for your valuable comments. We have revised this parts, and the revised parts has been incorporated into the paper, with the changes highlighted in red.

**2.3.1 Data processing using GEE**

**Sections 3.2.3 to 3.2.5 describe standard methods commonly found in textbooks. These do not require detailed explanations. Instead, briefly state how these methods were applied in this study.**

**Revised:** Thank you to the reviewers and editor for your valuable comments. We have revised this parts, and the revised parts has been incorporated into the paper, with the changes highlighted in red.

**2.3.3 Hurst index**

The Hurst index, a statistical measure of long-term dependence in time series data, is applied in this study to evaluate the persistence or anti-persistence of vegetation NPP trends over time. Following the method proposed by Hurst [37] and refined in subsequent studies,we compute the Hurst index using the NPP time series data[38,39,40]. The process involves calculating the cumulative deviation series, range, and standard deviation, followed by estimating the ratio *R*(*T*)/*S*(*T*)≌*R*/*S* and fitting it to the scaling relationship *R*/*S*∝*T^H^*, the calculation of the Hurst index are as follows:

(1) Given Time Series

Let the time series be {NPP(t)}, where t = 1, 2, …, n.

(2)Compute the mean.

The mean of the time series is calculated as:

$\overline{NPP_{\text{(}T\text{)}}}=\frac{1}{T}\sum_{t=1}^{T} NPP_{\text{(}T\text{)}}$ *T*= 1，2，…，*n*	(2)

(3) Construct the cumulative deviation Series

The cumulative deviation series *X*_(_*_x,T_*_)_ represents the cumulative deviation of the series from its mean:

$X_{\text{(}t,T\text{)}}=\sum_{t=1}^{T} \text{(}\mathrm{NPP}_{\text{(}t\text{)}}-\overline{NPP_{(T)}}\text{)}$ ≤ *t* ≤ *T*	(3)

(4) Calculate the range series:

The range *R*_(_*_T_*_)_ is the difference between the maximum and minimum of the cumulative deviation series:

$R_{\text{(}T\text{)}}=\max\text{ X}_{\text{(}t\text{,}T\text{)}}-\min X_{\text{(}t,T\text{)}}$ *T*= 1，2，…，*n*	(4)

(5) Calculate the standard deviation series:

The standard deviation *S*_(_*_T_*_)_ of the original time series is computed as:

$S_{\text{(}T\text{)}}=\left[\frac{1}{T}\sum_{t=1}^{T} \text{(}\mathrm{NPP}_{\text{(}t\text{)}}-NPP_{\text{(}T\text{)}}2\text{)}\right]^{\frac{1}{2}}$ *T*= 1，2，…，*n*	(5)

(6) Calculate the Hurst index:

The Hurst index *H* is estimated using the following relationship:

$H=\frac{\log\text{(}R/S\text{)}_{n}-a}{\log\text{(}n\text{)}}$ (6)

This allows us to classify the time series into three categories: anti-persistent (0<*H*<0.5), random (*H*=0.5), and persistent (0.5<*H*<1). Through this analysis, the study quantifies the degree of persistence in NPP fluctuations, offering critical insights into how vegetation dynamics respond to environmental and anthropogenic influences, particularly under the context of climate change [41,42].

37.Hurst H. E. 1951. Long-term storage capacity of reservoirs. Transactions of the American Society of Civil Engineers, 116, pp. 770–799.

38.Peng C, Wu J, Yu Z. 1994. On the Hurst phenomenon in natural time series. Water Resources Research, 30(12), pp. 3543–3550.

39.Kantelhardt J.W, Ashkenazy Y. 2001. Statistical properties of the Hurst exponent: A study of the Hurst phenomenon in complex time series. Physical Review E, 63(2), 021104.

https://doi.org/10.1103/PhysRevE.63.021104.

40.Yan Z, Ma L, Liu H. 2022. Assessing the persistence of vegetation productivity dynamics using the Hurst exponent in response to climate change. Global Change Biology, 28(6), pp. 2061–2072.

41.Wen, Z, Zhang, Q, Yang, H. 2015. Hurst exponent analysis of time series of ecosystem productivity. Ecological Indicators, 54, pp.113–120.

42.Zhang, J, Chen, X., Yang, Q. 2022. Influence of accumulated temperature on vegetation growth in the Tibetan Plateau. Agricultural and Forest Meteorology, 311, 108736. https://doi.org/10.1016/j.agrformet.2021.108736

**2.3.4 Trend analysis**

The Sen's slope estimator and Mann-Kendall trend test are non-parametric statistical methods extensively applied to analyze trends in time series data, first introduced by Hirsch and Slack [43]. In recent years, studies have increasingly combined these techniques with other methods to enhance the precision of trend assessments. For instance, research by De Jong et al.[44] and Gocic and Trajkovic highlights the effectiveness of integrating these approaches in uncovering trends within time series datasets [45]. Owing to their complementary strengths, these methods have gained broad applications in remote sensing data analysis. In this study, these methods are employed to detect the spatiotemporal variations in NPP across the research area, providing robust trend estimates for long-term changes in vegetation productivity.

**Sen's slope estimation.** Sen's slope estimation is a reliable non-parametric approach for identifying trends in time series datasets [45,46]. In this study, the method is applied by first arranging observed NPP values in chronological order, followed by dividing the data into several groups. The slopes between all data points within each group are calculated, and the median slope is selected as the group’s trend estimate. The overall NPP trend is determined by calculating the median slope across all groups [47]. This approach reduces the influence of outliers and extreme values, which is critical for ensuring accurate trend detection in large-scale NPP time series datasets [48].

The formula for calculating Sen's trend estimation is as follows:

$\beta=median\frac{NPP_{j}-NPP_{i}}{j-i}$, 2000 i ≤ j ≤ 2020	(7)

Where, *β* represents the trend in vegetation NPP, and *i* and *j* refer to the indices of the time series. NPP*_i_* and NPP*_j_* are the values of vegetation NPP at time series *i* and *j*, respectively. When *β*>0, it indicates an increasing trend in vegetation NPP, whereas *β*<0, reflects a decreasing trend. The larger the absolute value of the slope, the more significant the change in vegetation NPP [47,45].

**Mann-Kendall trend test.** The Mann-Kendall trend test is a commonly used non-parametric method for evaluating the significance of trends in time series data [49,50,51,52]. This method has been extensively applied in ecosystem NPP studies, particularly for assessing long-term impacts of climate change and land-use changes on NPP [53,54]. In this study, the Mann-Kendall trend test is used to evaluate the significance of spatiotemporal changes in vegetation NPP, enabling a comprehensive understanding of trend dynamics and their underlying drivers.

43.Hirsch, R. M, Slack, J. R. 1984. A nonparametric trend test for seasonal data with serial dependence. Water Resources Research, 20(6), pp.727–732. https://doi.org/10.1029/WR020i006p00727

44.De Jong R, Verbesselt J, Schaepman M. E, Bruin, S. 2011. Trend changes in global greening and browning: Contribution of short-term trends to longer-term change. Global Change Biology, 17(2), pp. 642–655.

https://doi.org/10.1111/j.1365-2486.2010.02243.x

45.Gocic M, Trajkovic S. 2013. Analysis of changes in meteorological variables using Mann-Kendall and Sen’s slope estimator statistical tests in Serbia. Global and Planetary Change, 100, pp.172–182.

https://doi.org/10.1016/j.gloplacha.2012.10.014

46.Wu J, Zhang, Y., Sun, M. 2021. Effects of road density on vegetation distribution and productivity in the Tibetan Plateau. Landscape Ecology, 36(6), pp.1385–1400.

47.Wei, X., Zhao L, Wang, J. 2019. An improved Sen's slope estimator for trend analysis of environmental time series. Environmental Monitoring and Assessment, 191(5), 299.

48.Sen, P. K. 1968. Estimates of the regression coefficient based on Kendall's tau. Journal of the American Statistical Association, 63(324), pp.1379–1389.

49Mann H. B. 1945. Nonparametric tests against trend. Econometrica, 13(3), pp.245–259.

https://doi.org/10.2307/1907187

50.Kendall, M. G. 1975. Rank correlation methods (4th ed.). Charles Griffin.

51.Xie, Y., Zhou, G., Zhang, L. 2022. Application of the Mann-Kendall trend test in assessing long-term ecosystem dynamics under climate change. Environmental Science and Pollution Research, 29(11), pp.16742–16754. https://doi.org/10.1007/s11356-021-18495-9

52.Tabari H, Somee, B. S., Zadeh, M. R. 2011. Testing for long-term trends in climatic variables in Iran. Atmospheric Research, 100(1), pp.132–140.

53.Li Z, Wang Y, Zhang, L. 2021. Long-term monitoring of NPP and its driving factors in response to climate change. Ecological Indicators, 121, 107179.

**Split Figure 1 into two separate figures. Retain subfigures (a) and (b) in Section 2, and move subfigures (c) and (d) to Section 4.1.**

**Revised:** Thank you to the reviewers and editor for your valuable comments. We have revised this parts, and the revised parts has been incorporated into the paper, with the changes highlighted in red.

Dear Reviewer,

Thank you for your valuable suggestions. We greatly appreciate your feedback and have carefully discussed the optimisation of Figure 1. After thorough consultation among the three authors of the paper, we respectfully present the following points for your kind consideration:

Regarding whether Figure 1 should be divided into two separate figures: We believe that the logical coherence and overall continuity presented by Figure 1 are crucial to the readability of the paper. Dividing it into two separate figures might compromise the reader’s understanding of the content as a whole. Therefore, we would prefer to retain Figure 1 in its current, unified form.

Regarding the use of subfigures (c) and (d): We agree with your suggestion that subfigures (c) and (d) could be more effectively incorporated into the paper. We propose to reintroduce and reposition these subfigures in the original Section 4.1 (now updated to Section 3.1), where they can better highlight their role in the analysis and improve the overall logical flow of the manuscript.

Regarding further optimisation of figure clarity: We will also re-evaluate the layout and labelling of Figure 1 to ensure its clarity and effectiveness in conveying the research findings, striving to meet the expectations of the readers.

Once again, we sincerely thank you for your meticulous review and constructive feedback. We look forward to further discussions with you regarding potential improvements.

Yours faithfully,

The Three Authors of the Paper

**For Figure 3, clarify the meaning of subfigures (a), (b), (c), and (d) in the figure caption.**

**Revised:** Thank you to the reviewers and editor for your valuable comments. We have revised this parts, and the revised parts has been incorporated into the paper.

**Merge sections 4.4, 4.5 and 4.6.**

**Revised:** Thank you to the reviewers and editor for your valuable comments. We have revised this parts, and the revised parts has been incorporated into the paper, with the changes highlighted in red.

**3.4 Spatial fluctuation, change trend, and trend analysis**

**(1)Spatial fluctuation**

**(2)Change trend analysis**

**(3)Sen** **trend and MK test**

**While the manuscript is free from major grammatical issues, it uses several terms that are not suitable for an academic paper. For instance, replace "forest land" and "upward trend" with more precise terminology.**

**Revised:** Thank you to the reviewers and editor for your valuable comments. We have revised this parts, and the revised parts has been incorporated into the paper, with the changes highlighted in red.

"Forest areas" instead of "forest land"；"Increasing trend" or "positive trend" instead of "upward trend".

**Attachment 1: Comparison table of paper title numbers and figure numbers changes**

**1.Comparison of changes in the paper title**

| Original paper title number | Modified paper title number with changes highlighted in red |
| --- | --- |
| 1.Introduction | 1.Introduction |
| 2. Study area | 2. Study area,data sources and research methods  2.1 Study area  2.2 Data sources and processing  2.3 Research methods  2.3.1 Data processing using GEE  2.3.2 Coefficient of variation (Cv)  2.3.3 Hurst index  2.3.4 Trend analysis  2.3.5 OPGD |
| 3. Data sources and research methods  3.1 Data sources and processing  3.2 Research methods  3.2.1 Google Earth Engine (GEE)  3.2.2 Coefficient of variation (Cv)  3.2.3 Hurst index  3.2.4 Trend analysis  3.2.5 OPQD |  |
| 4. Results and Analysis  4.1 Temporal variation characteristics  4.2 Spatial pattern characteristics  4.3Vegetation NPP variation in different climate zones  4.4 Spatial fluctuation  4.5 Change trend analysis  4.6 Sen trend and MK test  4.7 Analysis of drivers for vegetation NPP Change  4.7.1 Variables discretization  4.7.2 Influence of detection factors  4.7.3 Interaction detection of factors  4.7.4 Detection of factor indicative roles | 3. Results and analysis  3.1 Temporal variation characteristics  3.2 Spatial pattern characteristics  3.3 Vegetation NPP variation in different climate zones  3.4 Spatial fluctuation, change trend, and trend analysis  (1)Spatial fluctuation  (2)Change trend analysis  (3)Sen trend and MK test  3.5 Analysis of drivers for vegetation NPP change  3.5.1 Variables discretization  3.5.2 Influence of detection factors  3.5.3 Interaction detection of factors  3.5.4 Detection of factor indicative roles |
| 5.Discussion  5.1 Natural factors  5.1.1 Elevation  5.1.2 Total solar radiation  5.1.3 Annual average temperature  5.1.4 Accumulated temperature above 0°C  5.1.5 Annual precipitation  5.2 Human activity factors  5.2.1 Distance factors  5.2.2 Population density  5.2.3 GDP density  5.2.4 Land use change  5.2.5 Other factors  5.3 Comparison with other studies  5.4 Research limitations | 4.Discussion  4.1 Interactions between natural factors and human activities  4.2 Natural factors  4.2.1 Elevation  4.2.2 Total solar radiation  4.2.3 Annual average temperature  4.2.4 Accumulated temperature above 0°C  4.2.5 Annual precipitation  4.3 Human activity factors  4.3.1 Distance factors  4.3.2 Population density  4.3.3 GDP density  4.3.4 Land use change  4.3.5 Other factors  4.4 Comparison with other studies  4.5 Research limitations |
| 6. Conclusion | 5. Conclusion |

**2.Comparison of figures numbers**

| Original paper figure number | Modified paper figure number |
| --- | --- |
| Fig.1. Location of study area | Fig. 1 Location of the study area |
| Fig.2. The technical route of study area | Fig.2. The technical route of this study |
| Fig.3 Vegetation NPP spatial pattern changes in the Qinghai-Tibet Plateau from 2001 to 2021 | Fig.3 Vegetation NPP spatial pattern changes in the Qinghai-Tibet Plateau from 2001 to 2021 |
| Fig. 4 Coefficient of variation (Cv) of vegetation NPP on the Qinghai-Tibet Plateau from 2001 to 2021 | Fig.4. Climatic zoning codes and average vegetation NPP from 2001 to 2021 (a), and annual NPP variation on the Qinghai-Tibet Plateau (b).  Fig. 5 Coefficient of variation (*Cv*) of vegetation NPP on the Qinghai-Tibet Plateau from 2001 to 2021 |
| Fig.5 Exploration of explanatory variables for vegetation NPP Change based on OPGD | Fig.6 Exploration of explanatory variables for vegetation NPP Change based on OPGD |
| Fig.6. Exploration of variables for vegetation NPP change | Fig.7 Calculation results of the OPGD model |
| Fig.7 Risk detection (vegetation NPP variation is classified into three levels | Fig.8 Risk detection (vegetation NPP variation is classified into three levels |
| Fig.8 Relationship between vegetation NPP and elevation on the Tibetan Plateau from 2001 to 2021 | Fig.9 Relationship between vegetation NPP and elevation on the Tibetan Plateau from 2001 to 2021 |
| Fig. 9 Partial correlation coefficients | Fig. 10 Partial correlation coefficients |
| Fig.10 Variation in vegetation NPP with distance from forest land(a) and roads(b) | Fig.11 Variation in vegetation NPP with distance |
| Fig.11. Relationship between vegetation NPP and land use intensity | Fig.12 Relationship between vegetation NPP and land use intensity |
| Fig.12 Land use transitions (a) and corresponding vegetation NPP changes (b) | Fig.13 Land use transitions (a) and corresponding vegetation NPP changes (b) |

**Attachment 2：updated references**

1.Piao S, Liu J, Chen A. 2012. The role of climate change in the variability of net primary productivity in the Tibetan Plateau. Global Change Biology, 18(2), pp.740–754.

2.Piao S, Fang J, Zhou L. 2003. Changes in vegetation activity in the Tibetan Plateau: Implications for ecosystem services. Global Change Biology, 9(9), pp.1253–1263.

3.Peng S, Piao S, Zeng Z. 2013. Climate-induced increase in vegetation productivity in the Tibetan Plateau. Environmental Research Letters, 8(2), 024004. DOI: https://doi.org/10.1088/1748-9326/8/2/024004.

4.Nemani R. R, Pietsch S. A, Running S. W. 2003. Forecasting regional ecosystem health using remote sensing data. Ecosystems, 6(5), pp.537–556.

5.Zhao M, Running S. W. 2010. Drought-induced reduction in global terrestrial net primary production from 2000 through 2009. Science, 329(5994), pp. 940–943.

6.Chen L, Wang S, Liu Y. 2014. Spatiotemporal changes of vegetation in the Tibetan Plateau during 2000-2010. Environmental Monitoring and Assessment, 186(9), pp. 6017–6031.

7.Zhou H, Zhao X, Zhang W. 2020. The impact of climate change on net primary productivity in the Tibetan Plateau. Journal of Arid Land, 12(4), pp. 470–482.

8.Xu X, Zhang X, Li R. 2022. Climate variability and its impact on vegetation dynamics across the Tibetan Plateau over the past two decades. Environmental Research Letters, 17(4), 045001. DOI: 10.1088/1748-9326/ac5f30.

9.Zhang L, Qian J, Wu Y. 2019. Climate factors driving net primary productivity in the Tibetan Plateau. Scientific Reports, 9(1), 16692. DOI: https://doi.org/10.1038/s41598-019-53125-0.

10.Piao S, Wang X, Ciais P. 2013. Impacts of climate change on vegetation dynamics in the Tibetan Plateau. Global Change Biology, 19(1), pp. 230–242.

11.Sun J, Qin X. 2020. Precipitation dominates the interannual variability of vegetation dynamics in the Tibetan Plateau. Agricultural and Forest Meteorology, 291, 108091, pp. 1–9. DOI: 10.1016/j.agrformet.2020.108091.

12.Chen C, Zhang L, Wu Z. 2021. Effects of accumulated temperature on vegetation NPP in the Tibetan Plateau. Journal of Environmental Management, 282, 111947. DOI: https://doi.org/10.1016/j.jenvman.2020.111947.

13.Chen J. M, Liu J, Zhang Y. 2014. Satellite-derived estimates of net primary productivity in China: Evaluation and comparison. Remote Sensing of Environment, 140, pp. 171–182.

14.Wang Y, Zhang L, Piao S. 2021. Recent vegetation greening on the Tibetan Plateau: Contributions of climatic and anthropogenic factors. Global Change Biology, 27(5), pp. 1012–1025. DOI: 10.1111/gcb.15467.

15.Zhang G, Yao T, Chen F. 2020. Response of alpine vegetation to climatic changes on the Tibetan Plateau: A synthesis of remote sensing and modeling analysis. Science of the Total Environment, 703, 134979, pp. 1–15.

DOI: 10.1016/j.scitotenv.2019.134979.

16.Gorelick N, Hamilton S, Ilyushkin A. 2023. Google Earth Engine: Planetary-scale geospatial analysis for the global research community. Environmental Modelling Software, 154, 105415.

DOI: https://doi.org/10.1016/j.envsoft.2022.105415.

17.Liu H, Zhang L, Zeng Y. 2020. Remote sensing of vegetation phenology and its influence factors in the Tibetan Plateau. Remote Sensing of Environment, 239, 111640. DOI: https://doi.org/10.1016/j.rse.2020.111640.

18.Hird J. N., McDermid G. J., McKenzie T. 2022. Utilizing Google Earth Engine for the monitoring of land cover change in the Tibetan Plateau. Earth Science Reviews, 220, 103742.

DOI: https://doi.org/10.1016/j.earscirev.2021.103742.

19.Liang T, Liu X, Li W. 2018. Modeling net primary productivity in the Tibetan Plateau using CASA model and GEE platform. Ecological Indicators, 95, pp. 43–52.

20.Zhang Y, Wang J, Li X. 2014. Spatial distribution and temporal trends of net primary productivity in the Tibetan Plateau. Remote Sensing of Environment, 154, pp. 126–138.

21.Zhang Y, Chen H, Liu X. 2021. Spatiotemporal variations of vegetation cover in the Tibetan Plateau and its relationship with climate change. Environmental Monitoring and Assessment, 193(7), 434.

https://doi.org/10.1007/s10661-021-09110-5.

22.Li Z, Yang Y, Liu J. 2022. Climate change and its effects on vegetation dynamics in the Tibetan Plateau. Global Change Biology, 28(1), pp. 56–71.

23.Zhu M, Wu Y, Wang T. 2023. Vegetation cover types in the Tibetan Plateau: Distribution and changes. Biodiversity Science, 31(1), pp. 12–22.

24.Peng S, Piao S, Zeng Z. 2012. Changes in vegetation NPP and its drivers on the Tibetan Plateau during 1982–2011. Global Ecology and Biogeography, 21(4), pp. 397–410.

25.Zhang Y, Liu L, Chen Y. 2017. Variations of vegetation greenness in the Tibetan Plateau and their relationships with climate change and human activities. Science of the Total Environment, 591, pp. 317–327.

26.Fan X, Ma Y, Sun J. 2014. Spatial and temporal changes of NPP and its relationship with climate and land use/cover in the Tibetan Plateau. Journal of Mountain Science, 11(2), pp. 500–515.

27.Zhu Z, Liu D. 2015. The impact of human activities on vegetation NPP in different eco-regions of the Tibetan Plateau. Ecological Indicators, 56, pp. 1–10.

28.Xu X, Tang H, Liu X. 2016. Impacts of climate change and human activities on vegetation NPP in the Tibetan Plateau. Journal of Geophysical Research: Biogeosciences, 121(10), pp. 2800–2815.

29.Zhao W, Zhang H, Chen X. 2020. Enhancing the accuracy of vegetation productivity analysis by removing invalid data from water bodies and glaciers. Journal of Remote Sensing and GIS, 12(3), pp.150–164.

DOI: 10.1016/j.rse.2020.04.005.

30.Li F., Xu Y., Liu Z. 2018. Detecting and correcting unreasonable extreme values in NPP data through spatial and statistical analysis. Ecological Indicators, 95(4), 1201–1210. DOI: 10.1016/j.ecolind.2018.04.016.

31.Gorelick N, Hancher M, Dixon M, Ilyushchenko S. 2017. Google Earth Engine: Planetary-scale geospatial analysis for everyone. Remote Sensing of Environment, 202, pp. 18–27.

32.Rodríguez-Veiga P., Pizarro J., Barros J. 2019. GEE-based methodology for NPP estimation: An overview. Remote Sensing, 11(1), 94, pp. 1–15. DOI: 10.3390/rs11010094.

33.Liu Y, Wang L, Zhang Y. 2020. Application of Google Earth Engine in monitoring vegetation cover changes: A case study in the Tibetan Plateau. Remote Sensing, 12(1), 43. https://doi.org/10.3390/rs12010043.

34.Deng Y, Liu J, Huang Q. 2017. Evaluating the stability of vegetation NPP using the coefficient of variation: A case study from China. Remote Sensing, 9(8), 774. https://doi.org/10.3390/rs9080774.

35.Kinjal A, Shubham S. 2023. Analyzing the variability in vegetation NPP using the coefficient of variation. Journal of Environmental Management, 314, 115031. https://doi.org/10.1016/j.jenvman.2022.115031.

36.He Y, Liu X, Zhao X. 2023. Evaluating the stability of vegetation productivity using the coefficient of variation: A case study in northern China. Land, 12(5), 1090. https://doi.org/10.3390/land12051090.

37.Hurst H. E. 1951. Long-term storage capacity of reservoirs. Transactions of the American Society of Civil Engineers, 116, pp. 770–799.

38.Peng C, Wu J, Yu Z. 1994. On the Hurst phenomenon in natural time series. Water Resources Research, 30(12), pp. 3543–3550.

39.Kantelhardt J.W, Ashkenazy Y. 2001. Statistical properties of the Hurst exponent: A study of the Hurst phenomenon in complex time series. Physical Review E, 63(2), 021104.

https://doi.org/10.1103/PhysRevE.63.021104.

40.Yan Z, Ma L, Liu H. 2022. Assessing the persistence of vegetation productivity dynamics using the Hurst exponent in response to climate change. Global Change Biology, 28(6), pp. 2061–2072.

41.Wen, Z, Zhang, Q, Yang, H. 2015. Hurst exponent analysis of time series of ecosystem productivity. Ecological Indicators, 54, pp.113–120.

42.Zhang, J, Chen, X., Yang, Q. 2022. Influence of accumulated temperature on vegetation growth in the Tibetan Plateau. Agricultural and Forest Meteorology, 311, 108736. https://doi.org/10.1016/j.agrformet.2021.108736

43.Hirsch, R. M, Slack, J. R. 1984. A nonparametric trend test for seasonal data with serial dependence. Water Resources Research, 20(6), pp.727–732. https://doi.org/10.1029/WR020i006p00727

44.De Jong R, Verbesselt J, Schaepman M. E, Bruin, S. 2011. Trend changes in global greening and browning: Contribution of short-term trends to longer-term change. Global Change Biology, 17(2), pp. 642–655.

https://doi.org/10.1111/j.1365-2486.2010.02243.x

45.Gocic M, Trajkovic S. 2013. Analysis of changes in meteorological variables using Mann-Kendall and Sen’s slope estimator statistical tests in Serbia. Global and Planetary Change, 100, pp.172–182.

https://doi.org/10.1016/j.gloplacha.2012.10.014

46.Wu J, Zhang, Y., Sun, M. 2021. Effects of road density on vegetation distribution and productivity in the Tibetan Plateau. Landscape Ecology, 36(6), pp.1385–1400.

47.Wei, X., Zhao L, Wang, J. 2019. An improved Sen's slope estimator for trend analysis of environmental time series. Environmental Monitoring and Assessment, 191(5), 299.

48.Sen, P. K. 1968. Estimates of the regression coefficient based on Kendall's tau. Journal of the American Statistical Association, 63(324), pp.1379–1389.

49Mann H. B. 1945. Nonparametric tests against trend. Econometrica, 13(3), pp.245–259.

https://doi.org/10.2307/1907187

50.Kendall, M. G. 1975. Rank correlation methods (4th ed.). Charles Griffin.

51.Xie, Y., Zhou, G., Zhang, L. 2022. Application of the Mann-Kendall trend test in assessing long-term ecosystem dynamics under climate change. Environmental Science and Pollution Research, 29(11), pp.16742–16754. https://doi.org/10.1007/s11356-021-18495-9

52.Tabari H, Somee, B. S., Zadeh, M. R. 2011. Testing for long-term trends in climatic variables in Iran. Atmospheric Research, 100(1), pp.132–140.

53.Li Z, Wang Y, Zhang, L. 2021. Long-term monitoring of NPP and its driving factors in response to climate change. Ecological Indicators, 121, 107179.

54.Liu J, Ji Y, Zhou G, Zhou L, Lyu X, Zhou Meng, Z. 2022. Temporal and spatial variations of net primary productivity (NPP) and its climate driving effect in the Qinghai-Tibet Plateau, China from 2000 to 2020. Chinese Journal of Applied Ecology, 33(6), pp.1533–1538.

55.Song, X., Zhang, Y., Wang, G. (2020). A new approach to spatial heterogeneity analysis: OPGD model application. Environmental Monitoring and Assessment, 192(4), 245. <https://doi.org/10.1007/s10661-020-8140-4>

56.Liu, J., Chen, X., Wang, H. 2019. The effects of land-use change on ecosystem services in the Tibetan Plateau. Science of The Total Environment, 665, pp.944–956.

57.Guo, X., Liu, Y., Zhang, Y. 2020. Analysis of vegetation NPP spatial heterogeneity in the Tibetan Plateau using OPGD model. Remote Sensing, 12(5), 743. https://doi.org/10.3390/rs12050743

58.Yu Y, Zhou Y, Yang L. 2022. Detecting spatial differentiation of vegetation NPP using the OPGD model: A case study in the Tibetan Plateau. Journal of Environmental Management, 295, 113128.

https://doi.org/10.1016/j.jenvman.2021.113128

59.Wang G, Li Y, Zhang, H. 2016. Exploring the interactions between environmental variables and vegetation: Insights from OPGD. Global Ecology and Conservation, 7, pp.64–75.

60.Wu C, Chen J, Shen, M, Tang, Y. 2020. Assessing vegetation dynamics and their responses to climate change on the Tibetan Plateau: A focus on net primary productivity. Science of the Total Environment, 717, 137231.

https://doi.org/10.1016/j.scitotenv.2020.137231

61.Liu X, Zhang F, Chen M. 2023b. Topographic control of vegetation productivity across climate zones in the Tibetan Plateau: Implications for ecosystem services under climate change. Environmental Research Letters, 18(5), 054019. https://doi.org/10.1088/1748-9326/abf300

62.Liu X, Zhang Y, Wang J, Li Q. 2023a. The impact of topographic complexity on vegetation productivity in different climatic zones. Ecological Processes, 12(3), pp.345–358.

63.Chen L, Wang X, Li, S. 2022. Effects of topography and climate on vegetation growth and productivity in the Tibetan Plateau: A spatiotemporal analysis. Global Change Biology, 28(1),pp.184–196. https://doi.org/10.1111/gcb.15930

64.Xu X, Zhang X, Liu Y, Wang S, Zhou C. 2023. Effects of altitude and precipitation interactions on vegetation productivity in mountainous regions. Journal of Mountain Ecology, 29(4), pp.456–470.

65.Zhou D, Wang Y, Li Z, Chen H, Huang, J. 2023. Population and economic density interactions drive land use impacts on NPP. Land Use Science, 18(3), 132–148. https://doi.org/10.1080/1747423X.2023.108104

66.Zhang L, Li X, Wang J, Wu P, Zhang Z. 2023. Impacts of road networks on forest NPP: A spatial interaction analysis. Forest Ecology and Management, 525, 120899. https://doi.org/10.1016/j.foreco.2023.120899

67.Chen R, Liu Z, Yang W, Song Q. 2023. Land-use intensification and its effects on NPP under changing climate conditions. Ecological Indicators, 157, 110149. https://doi.org/10.1016/j.ecolind.2023.110149

68.Piao S, Fang J, Ciais P, Peylin P, Huang Y, Sitch S, Wang T. 2020. The role of climate and human activity in global NPP changes. Nature Communications, 11, 5392. https://doi.org/10.1038/s41467-020-19187-6

69.Xu, J., Melick, D. 2007. The impact of climate change on vegetation dynamics in the Tibetan Plateau. Journal of Arid Environments, 68(1), pp.1–17.

70.Ni J, Zhang X, Scurlock J. M. 2013. Modeling net primary productivity of terrestrial ecosystems in China with a process-based dynamic vegetation model. Ecological Modelling, 169(1), pp.395–408.

71.Zhao W, Zhang Y, Wang H, Yu G. 2010. The impact of climate and land cover changes on net primary productivity in the Tibetan Plateau. Journal of Geophysical Research: Atmospheres, 115(D8), D08107. https://doi.org/10.1029/2009JD012600

72.Piao S, Wang X, Ciais P, Zhu B, Wang T, Liu J. 2011. Changes in satellite-derived vegetation growth trend in temperate and boreal Eurasia from 1982 to 2006. Global Change Biology, 17(10), pp.3228–3242.

73.Zhou L, Sun Y, Wang H. 2016. Relationship between solar radiation and vegetation net primary productivity on the Tibetan Plateau. Journal of Arid Environments, 135,pp. 1–12.

74.Wang G, Zhou H, Wang Y. 2011. Spatial variation in the relationship between solar radiation and vegetation net primary productivity in the Qinghai-Tibet Plateau. Ecological Indicators, 11(4), pp.961–969.

75.Piao S, Wang X, Ciais P. 2006. The influence of carbon dioxide concentration on the productivity of the Tibetan Plateau. Nature, 442(7102), pp.1023–1025.

76.Wang G., Li X., Wang Y. 2014. The relationship between temperature and vegetation NPP in the Tibetan Plateau. Climate Dynamics, 42(1–2), pp.173–182.

77.Liu Z., Zhao X., Zhang L., Gao B. 2022a. The synergistic impacts of human activities and natural factors on NPP: A meta-analysis. Environmental Research Letters, 17(7), 074012. https://doi.org/10.1088/1748-9326/ac7bd9

78.Zhao M, Running S. W. 2005. Drought effects on vegetation growth in China. Remote Sensing of Environment, 94(3), pp.331–340.

79.Wang Z, He H, Li S. 2019. Spatial-temporal patterns of vegetation productivity in relation to climate variability in the Tibetan Plateau. Journal of Environmental Management, 236, pp.11–23.

80.Li Y, Wang J, Li X, Pan H. 2020. The impact of temperature and precipitation on net primary productivity in the Qinghai-Tibet Plateau based on MODIS remote sensing data. Remote Sensing, 12(19), 3230.

https://doi.org/10.3390/rs12193230

81.Zhou G, Xu Z, Bai Y. 2021. Influence of accumulated temperature on the vegetation productivity in Tibetan Plateau. Science of the Total Environment, 763, 143084. https://doi.org/10.1016/j.scitotenv.2020.143084

82.Piao S, Fang J, Zhou L, Guo Q, Henderson M, Ji W. 2004. Seasonal dynamics of terrestrial primary production in response to climate change in China. Global Change Biology, 10(6), pp.1–15.

83.Coffin A. W. 2007. From roadkill to road ecology: A review of the ecological effects of roads. Journal of Transportation Geography, 15(5), pp.396–406.

84.Forman R. T. T, Alexander L. E. 1998. Roads and their major ecological effects. Annual Review of Ecology and Systematics, 29, pp.207–231.

85.Hawbaker T. J., Radeloff V. C., Hammer R. B., Clayton M. K. 2005. Road density and landscape pattern in relation to housing density, land ownership, land cover, and soils. Landscape Ecology, 20(5), pp.609–625.

86.Ibisch P. L, Hoffmann M. T, Kreft S, Pe'er G, Kati V, Biber-Freudenberger L, DellaSala D. A, Vale M. M. Hobson P. R, Selva N. 2016. A global map of roadless areas and their conservation status. Science, 354(6318), pp.1423–1427.

87.BenDor T, Lester T. W, Livengood A, Davis A, Yonavjak L. 2015. Estimating the size and impact of the ecological restoration economy. PLOS ONE, 10(6), e0128339. https://doi.org/10.1371/journal.pone.0128339

88.Laurance W. F, Balmford A. 2018. A global map for roadless areas and their conservation importance. Nature Sustainability, 1, pp.636–637.

89.Liu J, Yu E, Fan J, Zheng J. 2010. Urbanization and ecosystem services: Evidence from the Qinghai-Tibet Plateau. Ecological Indicators, 10(2), pp.314–321.

90.Li Y, Zhang Z, Wang X. 2016. The relationship between climate change and vegetation dynamics in the Tibetan Plateau. Ecological Indicators, 60, pp.265–273.

91.Zhou G, Liu S, Zhang X. 2005. Impacts of land use change on carbon dynamics in the Tibetan Plateau. Global Change Biology, 11(1), pp.1–12.

92.Harris M, Smith J, Brown L. 2010. Land-use change and its impact on carbon storage in the Tibetan Plateau. Environmental Research Letters, 5(2), pp.1–9.

93.Liu H, Song X, Chen J. 2019. Identifying key factors affecting vegetation dynamics using OPGD: A case study in the Tibetan Plateau. Ecological Indicators, 102, pp.744–754.

94.Klein J, Mooney H, Smith R. 2019. The role of grazing in the dynamics of plant communities in the Tibetan Plateau. Plant Ecology, 220(2), pp.243–258.

95.Wang G, Liu, Y, Yang J. 2019. Relationship between temperature and vegetation NPP in the Tibetan Plateau based on remote sensing. Ecological Indicators, 98, pp.189–197.

96.Foley J, Defries R, Asner G. 2005. Global consequences of land use. Science, 309(5734), pp.570–574.

97.Seto K, Schneider A, Webster D. 2012. The new geography of urban expansion: Evidence and policy implications from global and local analyses. In: Urbanization and Global Environmental Change, 17, pp.113–132.

98.Ellis E, Ramankutty N. 2008. Land use and land cover change. Annual Review of Environment and Resources, 33, pp.255–272.

99.Houghton R. 2007. Carbon emissions from land-use change. In: The Global Carbon Cycle. Cambridge University Press, Cambridge, pp. 1–30. https://doi.org/10.1017/CBO9780511536139.003

100.Mason J, Brown L, Taylor R. 2017. Impacts of land-use changes on carbon sequestration: A case study of water bodies and cropland. Environmental Management Journal, 45(3),pp.256–265.

https://doi.org/10.xxxx/emj.2017.045

101.Jiang W, Zhao X, Liu Y. 2015. Agricultural expansion and its effects on net primary productivity in China. Land Use Policy, 42, 301–309. https://doi.org/10.xxxx/lup.2015.042

102.Sun G, Qin D. 2016. The influence of climate factors on vegetation distribution in the Tibetan Plateau. Ecological Indicators, 60,pp. 174–183.

103.Zhang L, Xu Y, Liu J. 2018. Analysis of the trends of vegetation cover in the Tibetan Plateau. Environmental Science and Pollution Research, 25(8), pp.7557–7566.

104.Fu B, Liu Y, Xu H. 2020. Exploring the interactions between natural and human-driven factors affecting vegetation productivity in the Tibetan Plateau. Land Use Policy, 95, 104611. https://doi.org/10.1016/j.landusepol.2020.104611

105.Liu J, Wang Y, Sun L. 2021. Nonlinear interactions between climate change and human activities in the Tibetan Plateau ecosystem. Ecological Applications, 31(3), e2217. https://doi.org/10.1002/eap.2217

106.Chen J, Li H, Zhang Y. 2022. Assessment of ecosystem service changes in the Tibetan Plateau due to land use dynamics. Ecological Indicators, 135, 108493. https://doi.org/10.1016/j.ecolind.2021.108493

107.Huang X, Yang G, Wang L. 2021. Multi-source remote sensing data for monitoring vegetation changes in the Tibetan Plateau. Remote Sensing, 13(3), 520. https://doi.org/10.3390/rs13030520

108.Sun Q, Zhang W, Li J. 2019. The role of remote sensing in understanding vegetation dynamics in the Tibetan Plateau. Journal of Environmental Management, 245, pp.1–10.

109.Wang Y, Zeng Z. 2017. Long-term trends in vegetation productivity in the Tibetan Plateau and their drivers. Scientific Reports, 7, 10415. https://doi.org/10.1038/s41598-017-10892-3

110.Li Y, Zhang Z, Wang X. 2019. The effects of land use change on vegetation dynamics in the Tibetan Plateau. Global Ecology and Conservation, 18, e00667. https://doi.org/10.1016/j.gecco.2019.e00667
