# Supplementary material for: Nonlinear variations and drivers of vegetation NPP on the Tibetan Plateau: Interaction of natural and human factors
Source: PLoS One. 2025 Jul 1;20(7):e0320370. doi: 10.1371/journal.pone.0320370 (PMC12212555; doi:10.1371/journal.pone.0320370)
Supplement: Attachment — Submitted filename: Response to Editors.docx [file pone.0320370.s005.docx]

**Response to Editors**

**Thanks editors for your comments, which will help improve the quality of paper.**

**To move forward, please review the proposed data availability statement below and confirm its accuracy. You can provide your confirmation in the author comments section, and we will update the statement accordingly.**

**Revised:** Thanks editors for your comments. We have provided the data availability statement indicating that all datasets are publicly available for research purposes, which has been highlighted in red and incorporated into the manuscript.

**Data availability statement**

We confirm that all datasets are publicly available for research use and verify their accuracy. The relevant datasets can be accessed through the provided links:

1. Vegetation NPP, soil moisture, evapotranspiration, and total solar radiation data are sourced from the Earth Engine data catalogue (https://developers.google.com/earth-engine/datasets).

2. China's administrative boundary data (1:4 million scale), natural factor data (including DEM, temperature, accumulated temperature ≥0°C, and precipitation), and human activity data (such as land use types, GDP density, and population density) are obtained from the Data Centre for Resources and Environmental Sciences, Chinese Academy of Sciences (RESDC) (http://www.resdc.cn).

3. Qinghai-Tibet Plateau boundary data are from the study "On the Extent and Area of the Qinghai-Tibet Plateau: Geographic Information System Data on the Boundaries and Area of the Qinghai-Tibet Plateau" published by Zhang et al. (2014) (http://www.geodoi.ac.cn/doi.aspx?doi=10.3974/geodb.2014.01.12.v1).
